# The *Tubotomaculum* Enigma and the Rise of Benthic Life During the Opening of the Western Mediterranean Basin

**DOI:** 10.1111/gbi.70031

**Published:** 2025-09-09

**Authors:** Simone Bernardini, Anas Abbassi, Paola Cipollari, Giancarlo Della Ventura, Cesareo Saiz‐Jimenez, Enrico Mugnaioli, Luigi Jovane, Armida Sodo, Fabio Bellatreccia, Mohamed N. Zaghloul, Domenico Cosentino

**Affiliations:** ^1^ Dipartimento di Scienze Università di Roma Tre Roma Italy; ^2^ Fachbereich Erdsystemwissenschaften Universität Hamburg Hamburg Germany; ^3^ Laboratory of Research and Development in Applied Geosciences (RDGA), FST‐Tanger University Abdelmalek Essaâdi‐Morocco Tétouan Morocco; ^4^ Instituto de Recursos Naturales y Agrobiologia (IRNAS‐CSIC) Sevilla Spain; ^5^ Dipartimento di Scienze della Terra Università di Pisa Pisa Italy; ^6^ Instituto Oceanográfico da Universidade de São Paulo São Paulo Brazil

**Keywords:** biomineralization, deep‐ocean ferromanganese nodules, extremophile microbial communities, fossil biofilms, permineralization, *Tubotomaculum*, Western Mediterranean Basin

## Abstract

Large‐scale geological processes shape microbial habitats and drive the evolution of life on Earth. During the Oligocene, convergence between Africa and Europe led to the opening of the Western Mediterranean Basin, a deep‐ocean system characterized by fluid venting, oxygen depletion, and the absence of benthic fauna. In this extreme, inhospitable seafloor environment, fusiform objects known as *Tubotomaculum* formed, whose origin has long remained controversial. We show that these enigmatic mineralizations consist of nanosized, poorly crystalline, phosphorus‐rich Mn‐Fe compounds produced through microbial mediation. They preserve carbonaceous material together with morphological, chemical, and mineralogical biosignatures, including high Mn oxidation state (3.9 ± 0.15), cell envelopes, extracellular polymeric substances (EPS), cell‐EPS partitioning of redox‐sensitive Mn and Fe, cluster‐assembled microbial cells, microbialite‐like and branching structures, and channel networks for nutrient transport. Geochemical signatures indicate precipitation under suboxic to anoxic, non‐sulfidic (post‐oxic) conditions from mixed seawater–hydrothermal fluids, with exposure on the seafloor prior to burial. The fusiform architecture of these self‐organized microbial populations suggests shaping by nutrient‐rich bottom currents associated with venting activity. This study provides a detailed glimpse into initial benthic colonization of the nascent Western Mediterranean Basin and establishes *Tubotomaculum* as a model for investigating biomineralization and microbial adaptation in extreme environments, with implications for the search for life beyond Earth.

## 1 Introduction

**FIGURE 1:**
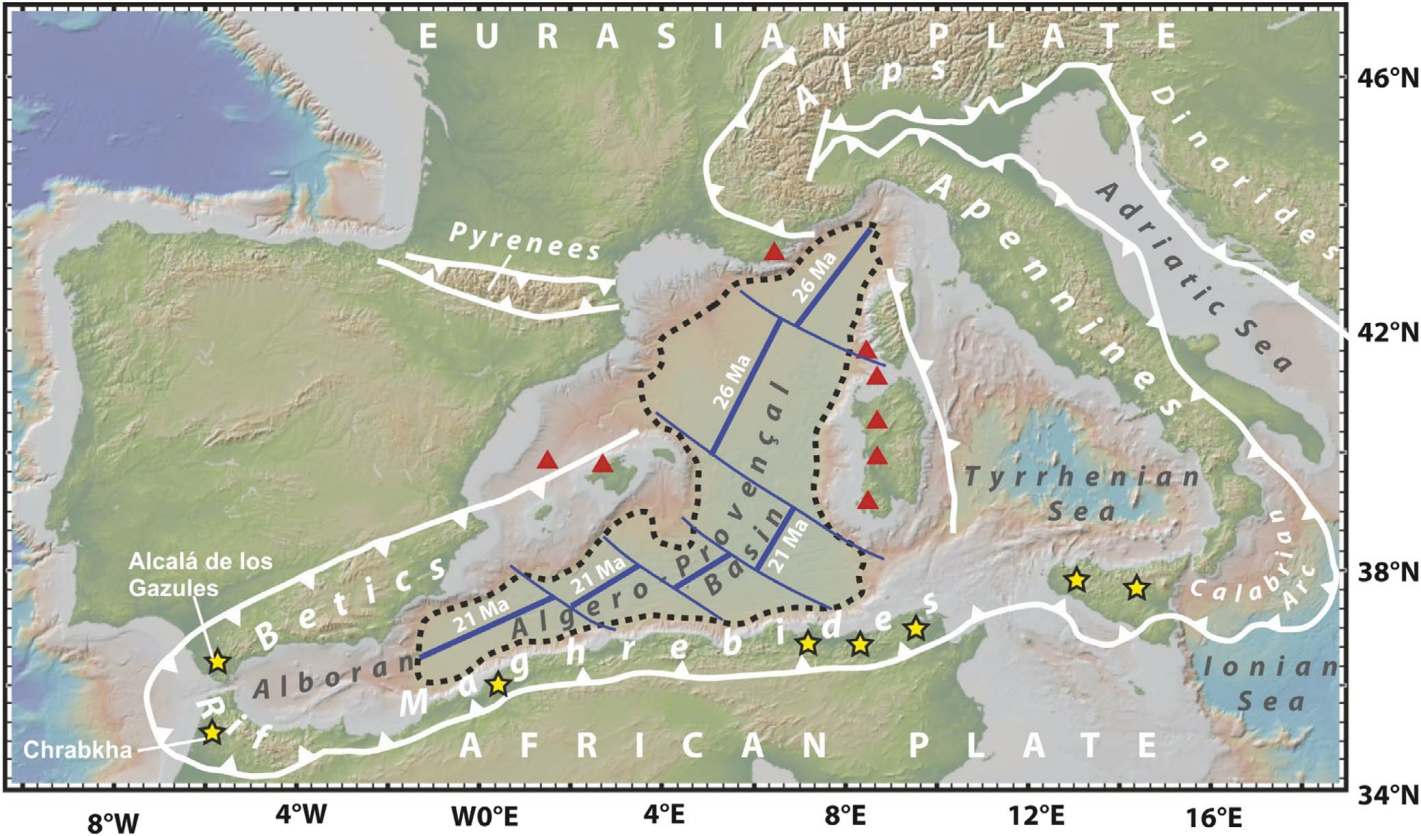
The birth of an ocean: The Western Mediterranean Basin. Schematic tectonic map of the Western Mediterranean subduction‐related orogen during the Oligocene. The blue thick line and associated age indicate the timing of the opening of the Western Mediterranean Ocean. Stars mark occurrences of *Tubotomaculum*. Triangles mark volcanic activity. Figure made with GeoMapApp (www.geomapapp.org)/CC BY/CC BY (Ryan et al. 2009).

The Western Mediterranean lies along a convergent plate margin separating Africa and Europe (Figure 1). During the Paleogene/Neogene transition, this boundary experienced interactions between orogenic processes and extensional tectonics. Beginning in the Oligocene (middle Chattian, ~26 Ma), intense extensional tectonics led to the formation of several back‐arc basins (e.g., the Ligurian Sea, the Algero‐Provençal Basin, the Valencia Trough, and the Alboran Sea). This process caused considerable thinning of the continental crust (e.g., the Alboran Sea) and initiated seafloor spreading, producing new oceanic crust (e.g., the Algero‐Provençal Basin, see Figure 1) (Rosenbaum et al. 2002; Schettino and Turco 2006; Carminati and Doglioni 2012; Savelli 2015; Gómez de la Peña et al. 2021). As a result, the region hosted deep‐water deposits that accumulated below the carbonate compensation depth (> 4000 m), forming the thick sequence known as Varicolored Clays (Guerrera et al. 2012; Riahi et al. 2014; García‐Ramos et al. 2014; Abbassi et al. 2021). These sediments (Oligocene‐Lower Miocene p.p.; Rupelian‐Aquitanian) accumulated in bathyal environments with severe oxygen depletion, as evidenced by the presence mainly of barren samples (de Capoa et al. 2007, 2014; Catalano et al. 2010; Carbone and Grasso 2012; Abbassi et al. 2021), which suggest the complete absence of benthic fauna.

**FIGURE 2:**
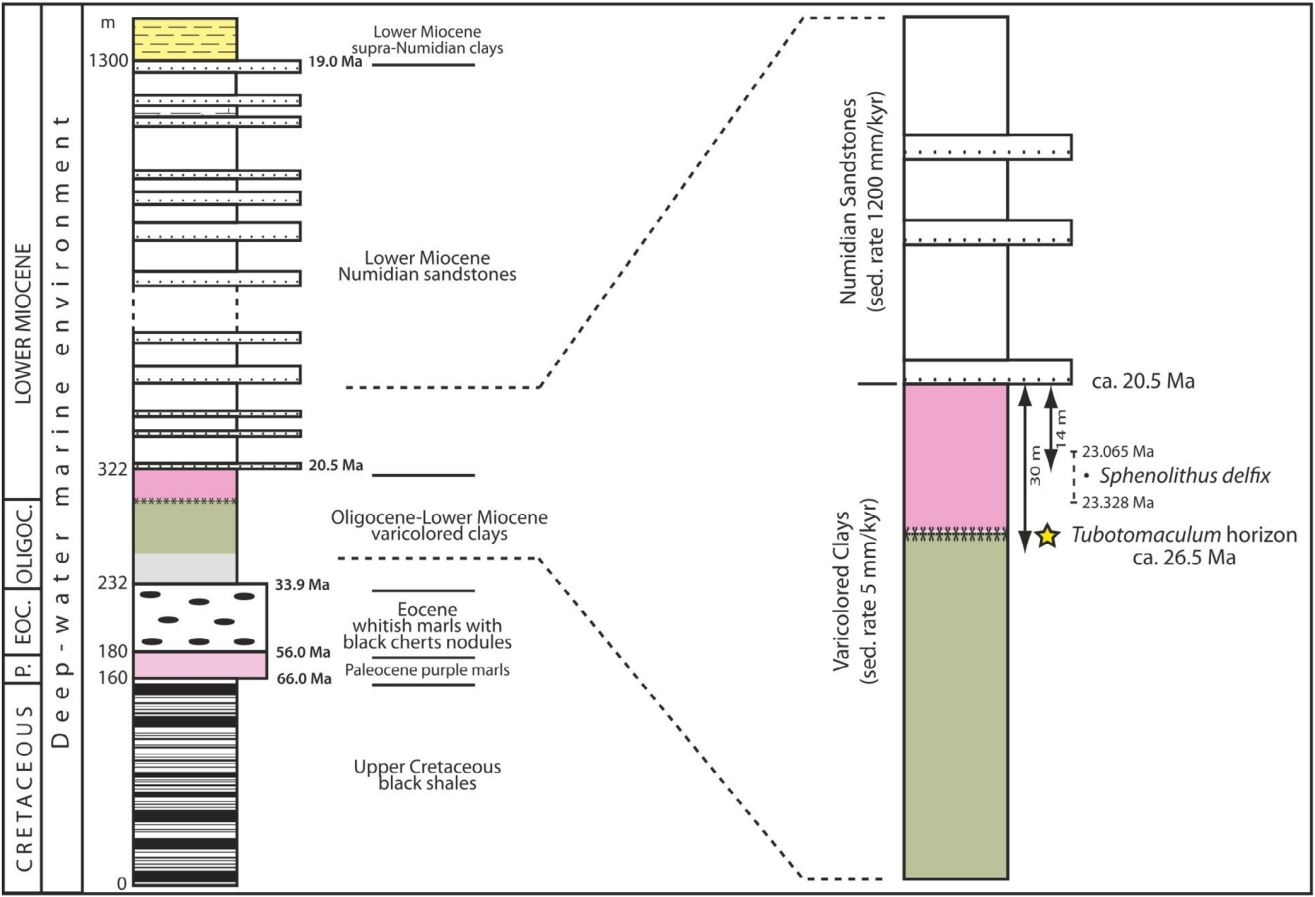
The *Tubotomaculum* horizon in the stratigraphic succession of the Western Mediterranean Basin. Stratigraphic log of the Chrabkha section (Tanger, Morocco, see Figure 1; Figures S2 and S4). Star marks the stratigraphic position of *Tubotomaculum*. After Abbassi et al. (2021).

In several localities around the Mediterranean (e.g., Spain, Morocco, Algeria, Tunisia, and Italy), the upper Varicolored Clays contain a distinctive horizon rich in enigmatic fusiform objects known as *Tubotomaculum* (star in Figure 2) (Durand‐Delga 1955; Pautot et al. 1975; García‐Ramos 1984; Hamoumi 2006; García‐Ramos et al. 2014; Riahi et al. 2014; Broquet 2016; Buatois et al. 2017; Uchman and Wetzel 2017; Menzoul et al. 2022; Naimi and Mahboubi 2025; Spadło et al. 2025). Since their first description, interpretations of *Tubotomaculum* have been both ambiguous and contradictory. The prevailing view, based solely on morphology, classifies them as ichnofossils (Durand‐Delga 1955; García‐Ramos 1984; García‐Ramos et al. 2014; Riahi et al. 2014; Buatois et al. 2017; Uchman and Wetzel 2017; Menzoul et al. 2022; Naimi and Mahboubi 2025; Spadło et al. 2025). Alternative hypotheses, however, include their interpretation as fossil corals encrusted with Mn‐Fe oxides (Hamoumi 2006), while geochemical studies have instead suggested an inorganic origin as polymetallic nodules precipitated directly from seawater (Pautot et al. 1975). The only consensus is that *Tubotomaculum* are composed of fine‐grained, poorly crystalline Mn‐Fe minerals, but their true origin remains unresolved.

Manganese and iron are the most abundant redox‐sensitive and biologically relevant metals on Earth. They occur in compounds with multiple oxidation states (Mn^2+^, Mn^3+^, Mn^4+^, and Fe^2+^, Fe^3+^) and diverse structural arrangements (e.g., Mn^*n*+^O_6_ or Fe^*n*+^O_6_ polyhedra arranged into channel or layered structures) that record environmental conditions of formation. Marine Fe‐Mn mineralizations are thus precious archives of past oceanic redox conditions, climate changes, bottom currents, continental erosion, and anthropogenic impact (Hein and Koschinsky 2014; Ortiz Kfouri et al. 2021; Bernardini et al. 2024; Cornaggia et al. 2020; Basilone et al. 2024). They can even preserve evidence of supernova events (Korschinek et al. 2020).

From a geochemical perspective, Mn remains soluble as Mn^2+^ at pH < 8, except under very high oxidation/reduction potential (Eh > 600 mV) (Hem 1972). Abiotic oxidation of Mn^2+^ to Mn^3+^/Mn^4+^ requires conditions unlikely to have prevailed in the oxygen‐depleted bottom waters of the forming Western Mediterranean Basin (WMB). Therefore, regardless of whether *Tubotomaculum* represents ichnofossils, fossil corals, or abiogenic nodules, their formation in such an extreme setting constitutes a genuine geological and geochemical enigma, implying that additional processes must have been involved.

Microbial activity could provide a plausible pathway. In most natural waters, Mn^2+^ oxidation is mediated by microorganisms, primarily bacteria and fungi, which produce poorly crystalline mixed‐valence Mn^3+^/Mn^4+^ oxides (e.g., vernadite, birnessite, todorokite, and buserite) with high average oxidation states (typically > 3.4) (Tebo et al. 2004, 2005). Microbes may catalyze Mn oxidation directly (via excreted polysaccharides or proteins) or indirectly (by altering the pH, redox conditions, or releasing oxidizing metabolites; Tebo et al. 2004, 2005). Crucially, biological Mn oxidation proceeds orders of magnitude faster than abiotic processes (Bargar et al. 2000; Tebo et al. 2004) and can occur at pH < 8 (Morgan 2005; Robbins and Corley 2005; Mao et al. 2023), allowing for Mn oxide formation in environments where abiotic processes are unfavorable. The occurrence of poorly crystalline Mn oxides in the geological record may therefore represent a biosignature (Bernardini, Bellatreccia, Columbu, et al. 2021). Deep‐sea microbial communities are capable of colonizing sediments even under severe nutrient and energy limitations (Jørgensen and Boetius 2007), thereby influencing the Mn and Fe cycling. Recent studies have indicated a biological contribution to abyssal Fe‐Mn nodules (Wang et al. 2012; Tully and Heidelberg 2013; Yli‐Hemminki et al. 2014; Blöthe et al. 2015) and Fe‐Mn crusts (Kato et al. 2018). By analogy, microbial colonization of the WMB seafloor during the Oligocene could have played a role in *Tubotomaculum* formation.

Despite decades of research, the mineralogical and chemical composition of *Tubotomaculum* remains poorly constrained, mainly due to the analytical challenges of characterizing fine‐grained, poorly crystalline Mn‐Fe mixtures using standard techniques. A multi‐method approach, combining diffraction techniques with vibrational spectroscopies (Raman and infrared) sensitive to both metal–oxygen bond strength and cation coordination, can overcome these limitations (e.g., Marino et al. 2019; Ortiz Kfouri et al. 2021; Cornaggia et al. 2020; Bernardini, Bellatreccia, Della Ventura, and Sodo 2021; Bernardini et al. 2024; Basilone et al. 2024). Interpreting such datasets within the framework of current genetic models for marine Fe‐Mn deposits (e.g., Bau and Dulski 1996; González et al. 2010, 2012; Marino et al. 2019; Hein et al. 2013; Hein and Koschinsky 2014; Bau et al. 2014; Ortiz Kfouri et al. 2021; Bernardini et al. 2024) offers the potential to reconcile morphological, mineralogical, and geochemical observations into a coherent genetic model.

In this study, we re‐evaluate the nature of *Tubotomaculum* using a comprehensive, multi‐method approach. Samples from the External Rif Chain (Morocco; Figure 1) were analyzed with optical microscopy (OM), scanning electron microscopy coupled with an X‐ray energy‐dispersive system (SEM‐EDS), X‐ray powder diffraction (XRPD), transmission electron microscopy (TEM), electron diffraction (ED), Fourier‐transform infrared (FT‐IR) spectroscopy, Raman spectroscopy (RS), and inductively coupled plasma mass spectrometry (ICP‐MS). These were complemented by synchrotron radiation‐based micro X‐ray fluorescence (SR‐μXRF) and X‐ray absorption near edge structure (SR‐XANES) spectroscopy. Following established biosignature identification protocols (Cady et al. 2003; Gillen et al. 2023), we examined features from the macro‐ to nanoscale, focusing on morphological, chemical, and mineralogical attributes diagnostic of microbial cells, cellular and extracellular processes, and carbonaceous remnants preserved during biomineralization. Based on this dataset, we propose a genetic model for *Tubotomaculum* that integrates stratigraphic, morphological, mineralogical, and geochemical evidence. Our aim is to assess whether these exotic mineralizations are biogenic or abiogenic and to clarify the processes driving Mn^2+^ oxidation on the seafloor of this extreme ocean system, thereby shedding new light on benthic colonization during the opening of the WMB.

## 2 Sampling Strategy

Two stratigraphic sections in the western Mediterranean (Spain and Morocco) comprising the *Tubotomaculum* horizon were sampled. The first section is located near Alcalá de los Gazules (Cádiz Province, South Spain; see Figure 1), where *Tubotomaculum* have previously been described by García‐Ramos et al. (2014) and García‐Ramos (1984). In this area, the *Tubotomaculum*‐bearing Varicolored Clays (“Arcillas con *Tubotomaculum*,” unit 16 of the Alcalá de los Gazules geological map; IGME 1984) are clearly part of a Lower Miocene tectono‐sedimentary complex. These Varicolored Clays incorporate abundant blocks of diverse lithologies and ages, including “Serie calcarea,” “Calizas bioclasticas y margas,” “Calcarenitas margosas y argillas rojas,” “Areniscas del Aljibe,” “Calizas (Bloque Jurassico),” “Caliza de Microcodium (Bloque Paleoceno),” “Areniscas tableadas (Bloque Oligocenico)” (Figure S1a), indicating significant reworking. The most frequent blocks derive from the “Areniscas del Aljibe” (Numidian Sandstones) (Figure S1b), suggesting that this tectono‐sedimentary complex formed after the deposition of the Numidian Sandstones (Lower Aquitanian). In the western Mediterranean, these sandstones systematically overlie the *Tubotomaculum* horizon. Therefore, in the Alcalá de los Gazules area, the *Tubotomaculum* horizon must be considered, without any doubt, as a reworked unit. In particular, our micropaleontological analysis of these clays revealed mixed fauna assemblages from the Upper Cretaceous, Palaeocene, and Eocene. In addition, typical Early Miocene taxa (e.g., *Globigerinoides* sp. and *Discoaster druggi*) were also found in some samples (IGME 1984). This fossil record thus reflects a chaotic mixture of geological materials from different ages (see, for example, Figure S1c). Consequently, samples from this area are unsuitable for accurately reconstructing the original paleoenvironment in which *Tubotomaculum* formed.

The second section is located in the External Rif Chain, northern Morocco, where the occurrence of *Tubotomaculum* has been documented by several authors (Hamoumi 2006; Abbassi et al. 2021). We selected the 9th April Dam section, which crops out near the Reservoir Lake 9th April in the western Rif area (Figure 1). The stratigraphic details are shown in Figure 2. This section represents a continuous succession from the Upper Cretaceous to the lower Burdigalian (Abbassi et al. 2021). In this sequence, the Varicolored Clays series (~90 m thick) is perfectly concordant, undeformed, and lacks allochthonous lithological blocks (Figure S2). The *Tubotomaculum* horizon occurs ~30 m below the Varicolored Clays/Numidian Sandstones transition (Figure 2) and consists of greenish clays at the base and reddish clays at the top (Figure S2).

**FIGURE 3:**
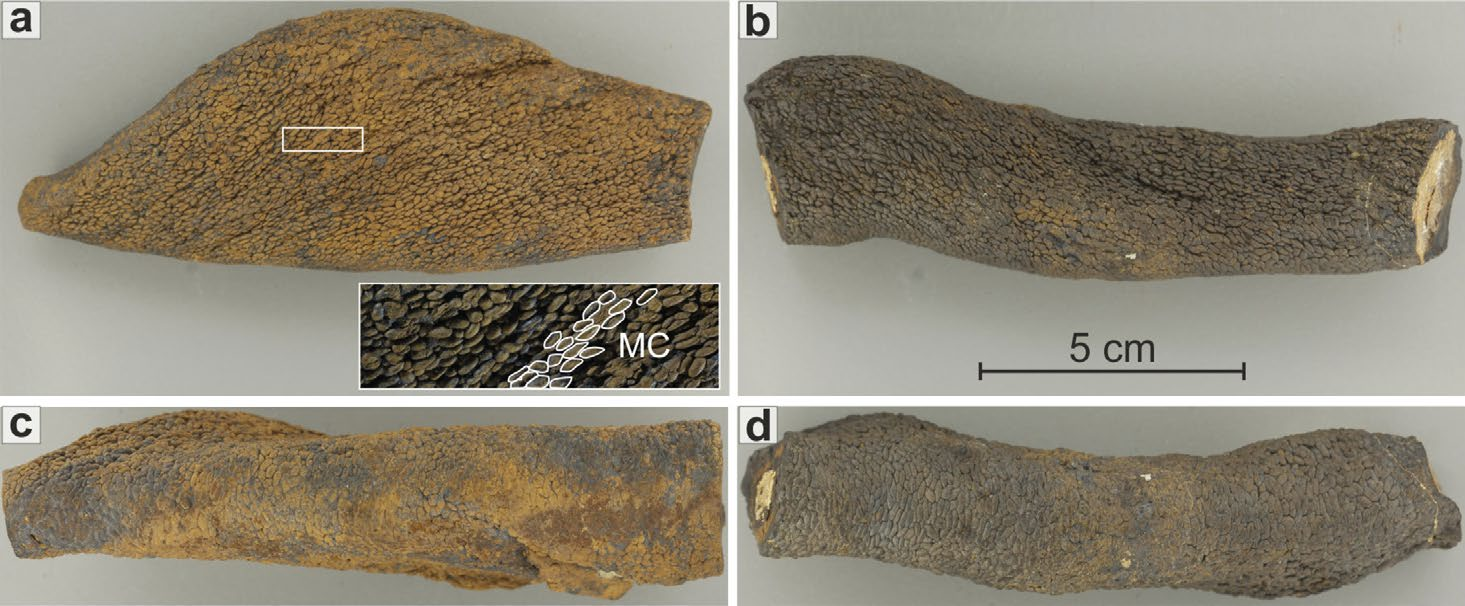
Three‐dimensional morphology of *Tubotomaculum*. Lateral (a, b) and bottom (c, d) views of two *Tubotomaculum* samples characterized by a fusiform shape. MC: Rice‐shaped grains interpreted as microbial‐like clusters. The scale bar is the same for all panels.

**FIGURE 4:**
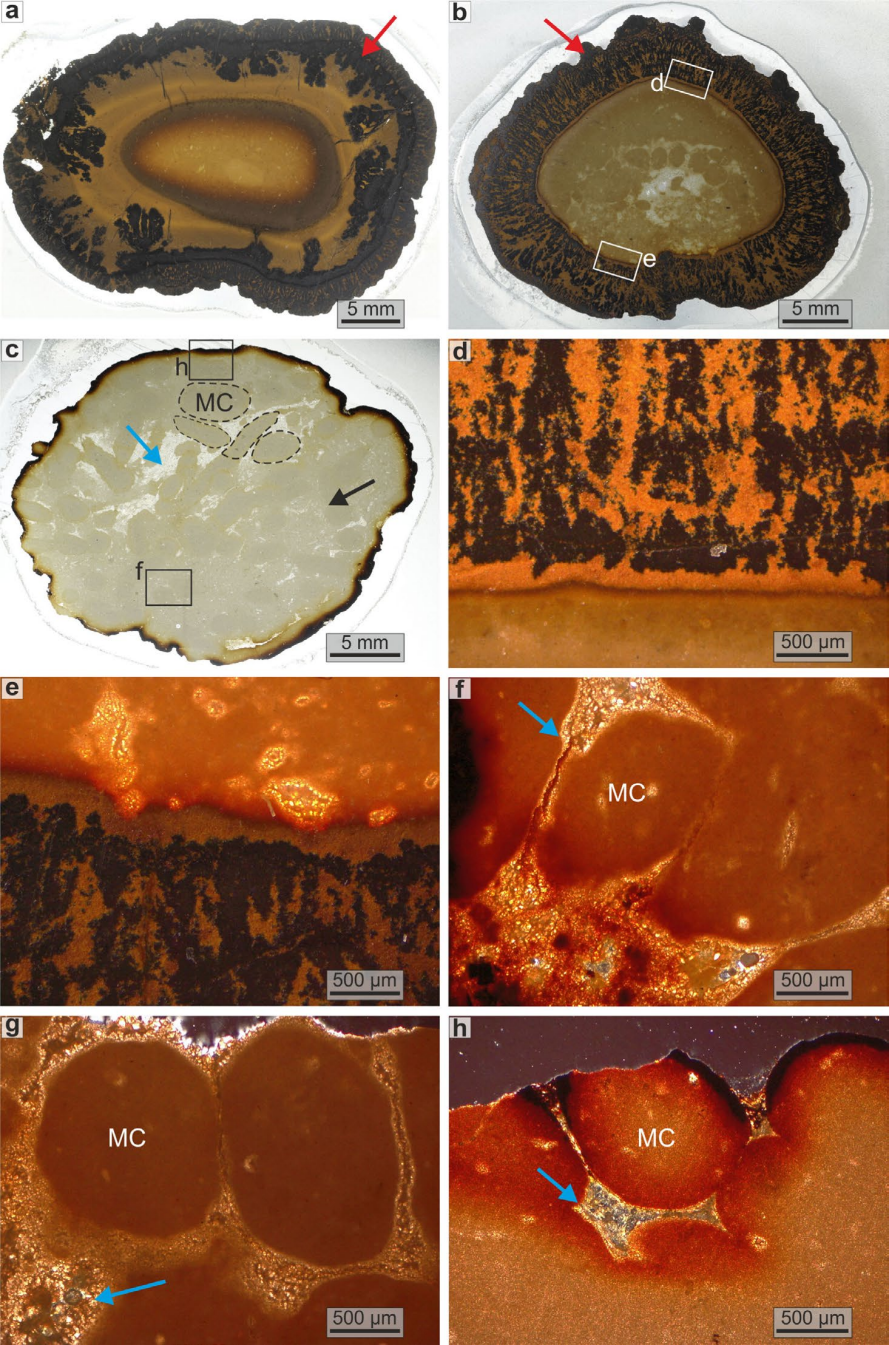
Internal structures of *Tubotomaculum*. Optical microscopy (OM) images of oxide‐dominant (a, b) and carbonate‐dominant (c) types. Details of the radial red filaments associated with channels in oxide‐dominant *Tubotomaculum* (d, e) and of microbial‐like clusters (MC) located inside a carbonate‐dominant sample (f, g) and at the outermost rim (h). Cyan arrows: Open‐water channels and voids, interpreted as a nutrient‐transporting channel network. Red arrows: Oxide‐rich external rim.

In outcrop, *Tubotomaculum* appear as isolated, horizontally oriented, blind‐ended nodule‐like mineralizations with a characteristic fusiform shape (see Figure 3; Figure S3a,b). Their surface is characterized by the presence of rice‐shaped grains ~1 mm in size (Figure 3). Sample size varies considerably, with lengths ranging from 1 to 10 cm and diameters from a few millimeters to ~4 cm (Figure 3). Based on stratigraphic position and macroscopic features, the specimens can be divided into two groups: (1) oxide‐dominant *Tubotomaculum*, consisting of a carbonate or detrital nucleus (i.e., fragments of spongy bone tissue and/or aggregates of quartz and phyllosilicates; Figure 4; Figure S3c) surrounded by an oxide‐rich external rim (red arrow in Figure 4a,b). These specimens occur within the reddish clays of the upper portion of the section (Figure S2). (2) Carbonate‐dominant *Tubotomaculum*, which consists of carbonate minerals coated by a blackish, fine‐grained oxide layer (Figure 4c). These occur within the greenish clays in the lower portion of the section (Figure S2).

## 3 Materials and Methods

Three oxide‐dominant specimens—Tub‐A (detrital nucleus with oxide rim; Figure 4a), Tub‐B, and Tub‐C (carbonate nucleus with oxide rim; Figure 4b)—and three carbonate‐dominant specimens (Tub‐Ca, Tub‐Ca1, and Tub‐Ca2; Figure 4c) were selected for mineralogical and geochemical analyses. In addition, 15 samples of the associated clays were collected for biostratigraphic analysis on both foraminifera and calcareous nannofossils.

Fragments from the oxide rims of samples Tub‐A, Tub‐B, and Tub‐C, and from carbonate‐dominant samples Tub‐Ca, Tub‐Ca1, and Tub‐Ca2, were hand‐picked under a binocular microscope. Selected material was ground to fine powders in an agate mortar for XRPD, FT‐IR, and SR‐XANES analyses. Polished thin sections were prepared by impregnating the samples with epoxy resin to preserve integrity during polishing for OM, SEM‐EDS, SR‐μXRF, and RS analyses. Fragments from both oxide‐dominant and carbonate‐dominant samples were analyzed by ICP‐MS.

A lamella from the external rim of the oxide‐dominant *Tubotomaculum* specimen Tub‐A was extracted and thinned to electron transparency by Focused Ion Beam (FIB) milling for TEM and ED.

### 3.1 Biostratigraphy Analysis

Samples for foraminiferal analyses (both benthonic and planktonic) were disaggregated in a 5% H_2_O_2_ solution, washed under tap water on 0.066‐ and 0.125‐mm mesh sieve sizes, and dried in an oven. Smear slides for light microscopic analysis of calcareous nannofossils were prepared using standard techniques. Abundances of selected calcareous nannofossil taxa were determined with a Zeiss Axioplan microscope (transmitted light and crossed nicols) at 1000× magnification.

### 3.2 Mineralogical and Chemical Analyses

SEM‐EDS data were collected using a Zeiss Sigma 300 FE‐SEM (field‐emission scanning electron microscope) equipped with a high‐resolution backscattered electron (HDBSE) detector and a Bruker QUANTAX 60 × 60 mm^2^ energy‐dispersive (EDS) detector. Elemental compositions were determined using an accelerating voltage of 20 kV and a filament current of 1.80 A.

XRPD data were collected using a Scintag X1 diffractometer under CuKα1 radiation (*λ* = 1.54055 Å, 40 mA, 45 kV), fixed divergence slits, and a Peltier‐cooled Si(Li) detector (resolution < 200 eV). A divergent slit width of 2 mm and a scatter‐slit width of 4 mm were employed for the incoming beam; a receiving slit width of 0.5 mm and a scatter‐slit width of 0.2 mm were used for the diffracted beam. Data were collected in step‐scan mode from 2° to 80° 2θ, with a step size of 0.05° 2θ and a counting time of 3 s per step.

Powder IR data were collected using a Nicolet iS50 FT‐IR spectrometer equipped with a DTGS detector and a KBr beamsplitter. The nominal resolution was 4 cm^−1^, and 64 scans were averaged. Samples were prepared as pellets containing ~1 mg of powdered sample in 200 mg of KBr.

Raman spectra were collected using an inVia Renishaw spectrometer equipped with a 532 nm diode laser (maximum output power 100 mW), an edge filter, an 1800 lines/mm diffraction grating, and a Peltier‐cooled 1024 × 256‐pixel CCD detector. Laser focusing and Raman signal collection were performed with a 50× long‐working‐distance objective. Spectra were acquired at 226 μW/μm^2^ with 30 s exposure to prevent laser‐induced degradation (Bernardini et al. 2020; Bernardini, Della Ventura, Mihailova, and Sodo 2025; Bernardini, Della Ventura, Sodo, and Mihailova 2025). Prior to measurements, the spectrometer was calibrated to the Si Raman peak at 520.5 cm^−1^. Data acquisition and analysis were performed using WiRE and OriginPro software; spectra were baseline‐corrected and fitted with pseudo‐Voigt functions to derive the phonon wavenumber and intensity. The spectral resolution was ±2 cm^−1^, and the instrumental precision for peak positions was ~0.5 cm^−1^.

SR‐XANES and SR‐μXRF analyses were performed at the XRF beamline of the Brazilian Synchrotron Light Laboratory (LNLS, Campinas, Brazil). XANES spectra were processed using the Athena package (Ravel and Newville 2005), with energy‐edge correction following Farges (2005). After normalization, pure Mn^3+^ and Mn^+4^ reference spectra were used for linear combination fitting to quantify Mn species. μXRF mapping was performed at 10 keV over an area of 6.2 × 1 mm^2^ (red dashed rectangle in Figure 10), at a 50 μm step size, for a total of 2646 points, with a counting time of 600 ms per point to determine Zn and Ni distribution. PyMCA 5.4.2 and OriginPro were used for calibration, processing, and data elaboration.

Transmission electron microscopy (TEM) and electron diffraction (ED) were conducted using a Zeiss Libra 120 TEM operating at 120 kV with a LaB_6_ source. TEM images were recorded with a TRS 2k × 2k camera, and polycrystalline ring‐like diffraction patterns were recorded with an ASI Timepix single‐electron camera and analyzed using ImageJ.

ICP‐MS analyses were performed at the Activation Laboratories Ltd. (Ontario, Canada). Each 0.25 g sample underwent four‐acid digestion: initial hydrofluoric acid followed by a nitric‐perchloric mixture, using ramp‐hold heating cycles to near dryness. Samples were re‐dissolved in aqua regia before ICP‐MS measurements. Quality control (QC) included 14% digestion checks per batch, 5 method reagent blanks, 10 in‐house controls, 10 duplicates, and 8 certified reference materials. Instrumental QC (13%) monitored drift during analysis. Because some elements occur in acid‐resistant minerals (e.g., zircon, monazite, sphene, xenotime, chromite, and barite), La, Ce, Nd, Sm, Yb, and Lu were additionally measured by Instrumental Neutron Activation Analysis (INAA). A 30 g aliquot was encapsulated in polyethylene, irradiated with flux wires at a thermal neutron flux of 7 × 10^12^ n cm^−2^ s^−1^, and cooled for 7 days to allow Na‐24 decay. The sample was counted using a high‐purity Ge detector (resolution better than 1.7 KeV for the 1332 KeV Co‐60 photopeak). Decay‐corrected activities, calibrated against multiple certified reference materials, yielded concentrations consistent with those obtained by ICP‐MS.

## 4 Results

### 4.1 Biostratigraphy

The biostratigraphic analysis showed that all clay samples from the *Tubotomaculum* horizon (both greenish and reddish clays; Figure S2) are barren. This result is consistent with Abbassi et al. (2021), who also reported barren samples and the absence of benthic fauna in the same Varicolored Clays studied here. The only species identified was *Sphenolithus delphix* (23.065–23.328 Ma; Raffi et al. 2006), found in a sample collected 14 m below the Varicolored Clays/Numidian Sandstones transition (see Figure 2). Barren samples and the absence of benthic fauna in the Varicolored Clays have also been documented in other western Mediterranean localities (de Capoa et al. 2007, 2014; Catalano et al. 2010; Carbone and Grasso 2012).

### 4.2 Morphological and Geochemical Characterization of *Tubotomaculum*

A preliminary OM study of the oxide‐dominant *Tubotomaculum* external rim (a few to several millimeters thick; red arrows in Figure 4) showed a layered texture of alternating black and red concentric layers, each up to several hundred microns thick (Figure 4a). These layers display microbialite‐like wrinkled laminations (Figure S5) and a radial network of red dendritic filaments reaching several millimeters in length (Figure 4b,d,e; Figure S5). In some samples, multiple erosion surfaces (green and red dotted lines) cut across the red and black (laminated or massive; 1 and 2 in Figure S3d–f) layers, producing relicts and angular unconformities where older layers are sharply truncated and overlain by younger ones (Figure S3e,f). In contrast, carbonate‐dominant *Tubotomaculum* (Figure 4c) consists of rice‐shaped grains (MC in Figure 4f–h) separated by a well‐developed network of voids and channels (cyan arrows in Figure 4).

**FIGURE 5:**
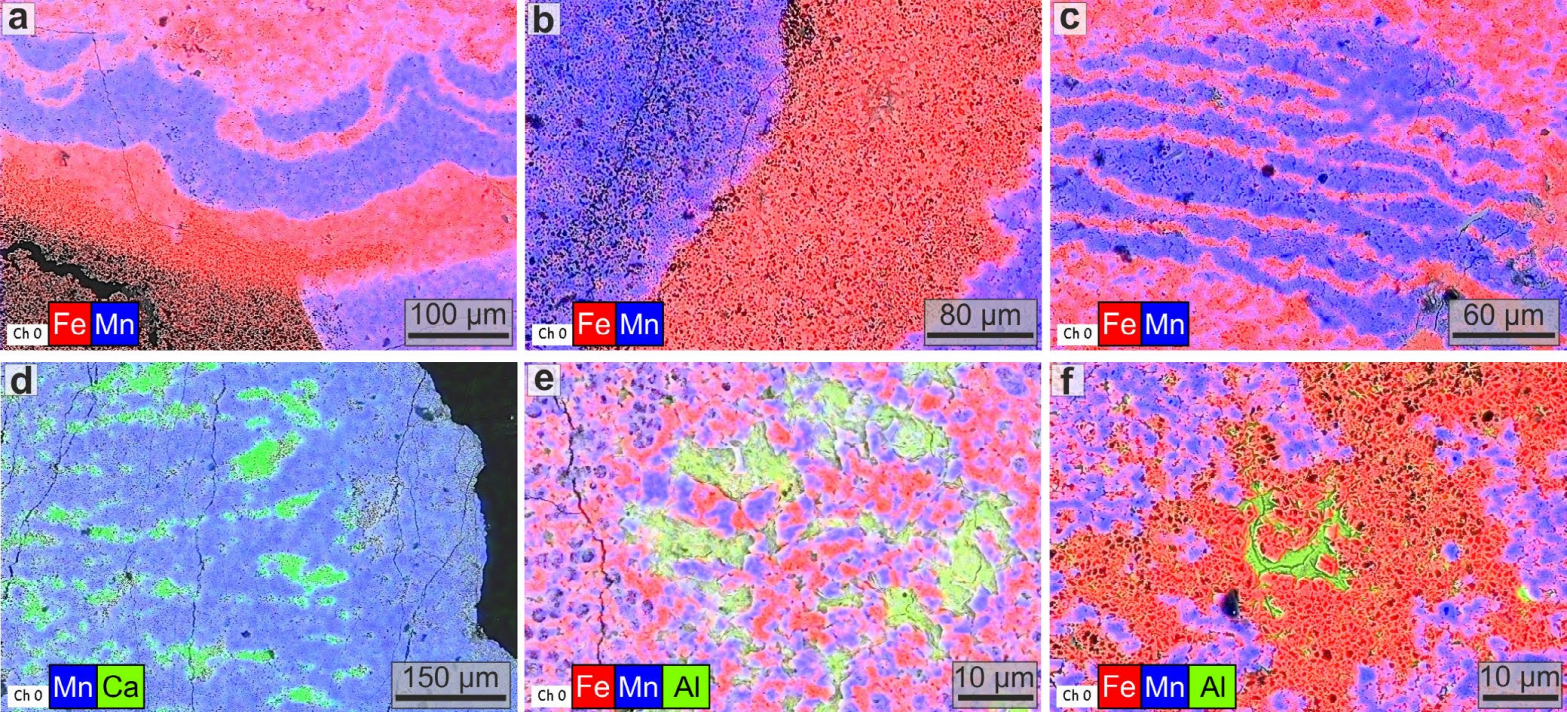
Spatial distribution of Mn and Fe at the microbial‐community‐level scale. EDS elemental maps showing that the layers and microbialite‐like structures in the inner portion (a–c) and the radial dendritic red filaments (d) in the outermost part of the oxide‐dominant *Tubotomaculum* (Figure 4a,b) result from the spatial distribution of Mn, Fe, and Ca. Al‐rich phyllosilicates within channels in oxide‐dominant *Tubotomaculum* (e, f).

**FIGURE 6:**
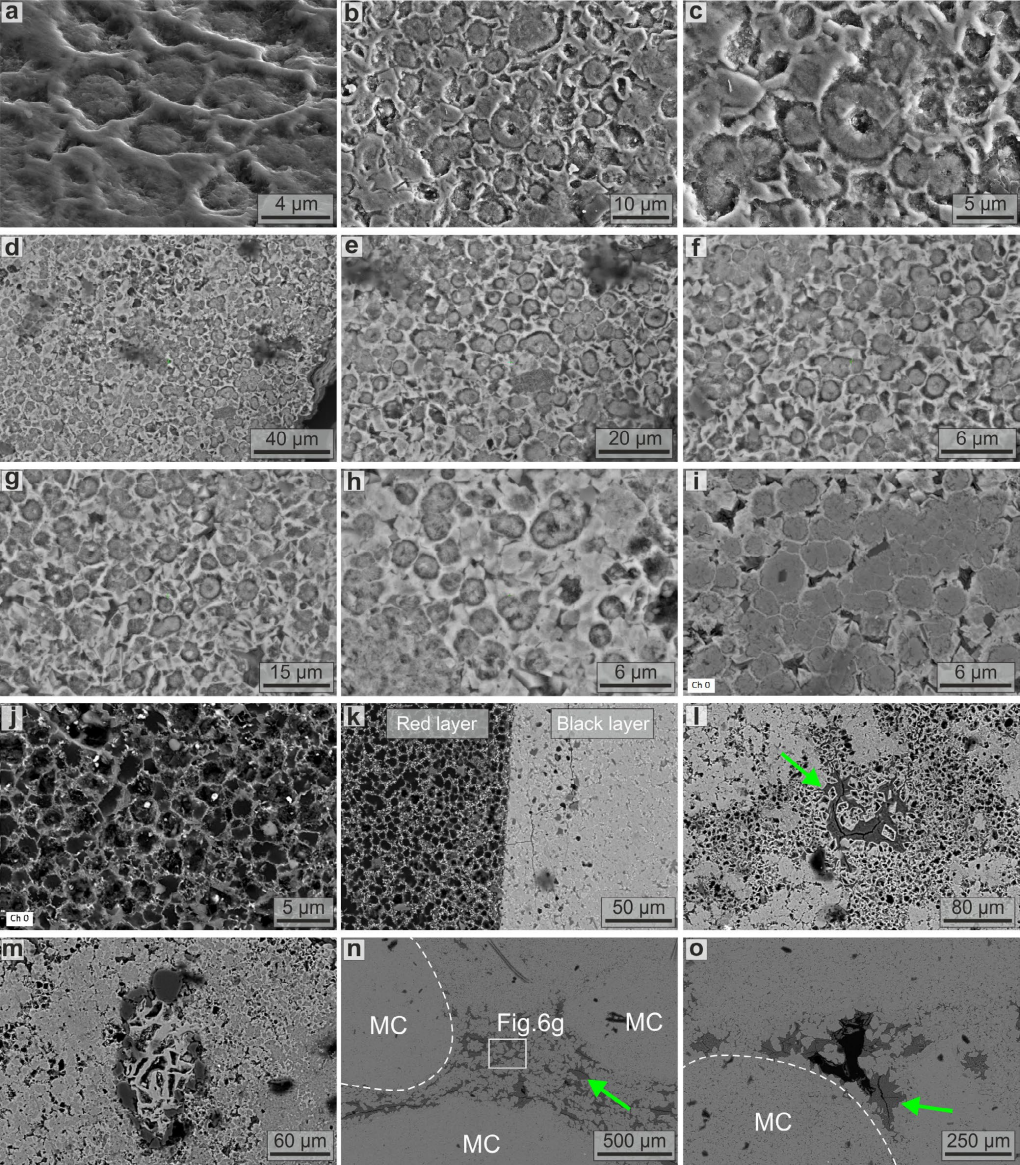
Internal structures of *Tubotomaculum* at the microbial‐cell scale. SEM images from black layers (a–i), brown layers (j), and their boundary (k) in oxide‐dominant *Tubotomaculum*. Images of channels and voids in oxide‐dominant (l, m) and in carbonate‐dominant *Tubotomaculum* (n, o). Green arrows: Phyllosilicate minerals filling the micro‐channels in oxide‐dominant *Tubotomaculum* and the voids between microbial‐like clusters (MC) in carbonate‐dominant *Tubotomaculum*.

**FIGURE 7:**
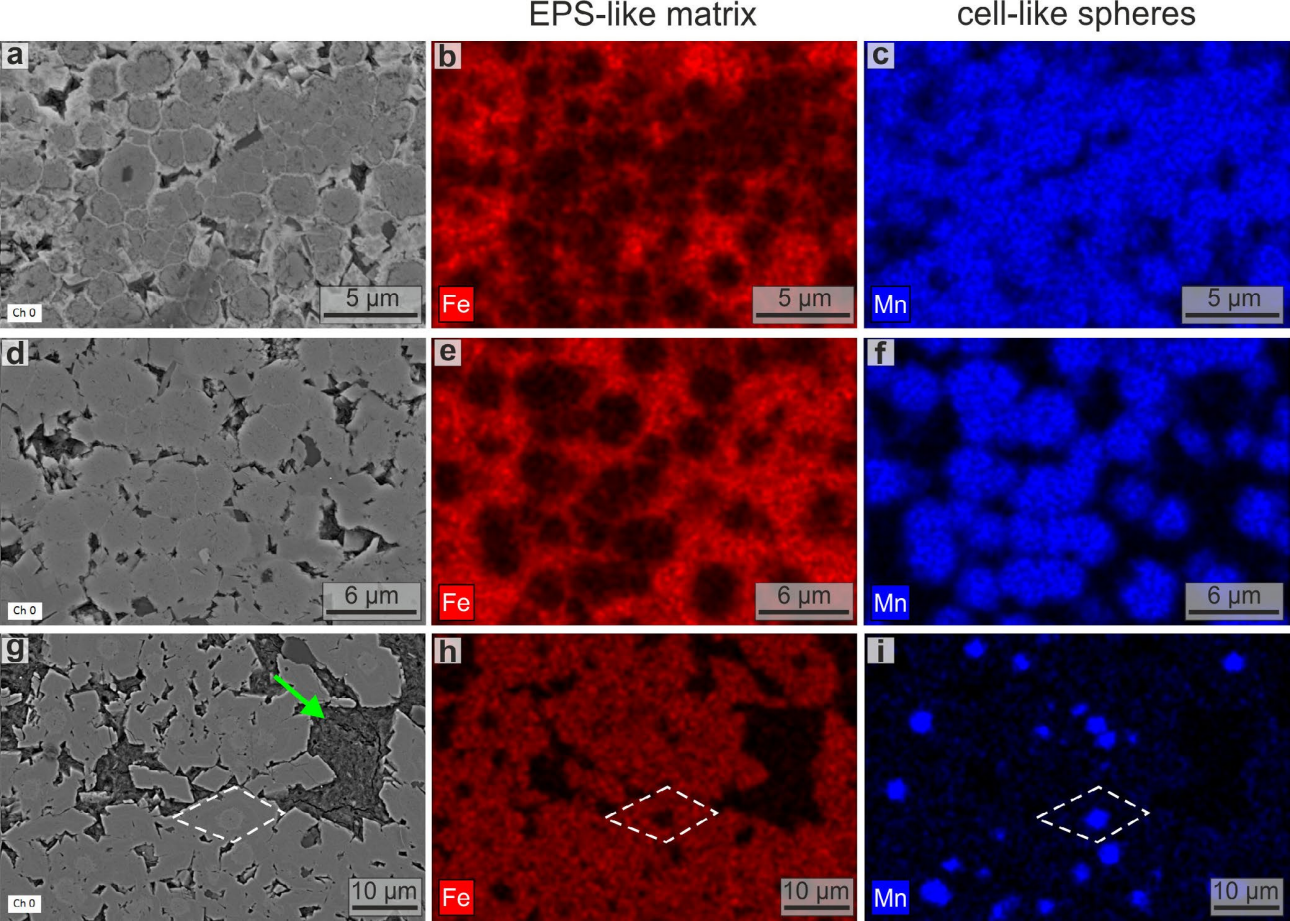
Spatial distribution of redox‐sensitive Mn and Fe at the microbial‐cell scale. EDS mapping of Mn and Fe in oxide‐dominant (a–c) and carbonate‐dominant (d–f) *Tubotomaculum*. Rhombohedral siderite crystals with rhodochrosite cell‐shaped cores occur in voids between microbial colonies (Figure 6n) in carbonate‐dominant *Tubotomaculum* (g–i). Green arrows: Phyllosilicate minerals. White dashed lines: Rhombohedral siderite crystal with a rhodochrosite cell‐shaped core, interpreted as the result of fluid circulation between the microbial‐like clusters.

SEM‐EDS imaging reveals a sharp separation between Mn and Fe that produces both the black/red concentric layers with microbialite‐like structures (Figure 5a–c; Figure S5) and the Fe‐Ca‐rich red dendritic filaments forming the channel network (Figure 5d). Many of these channels are infilled with Al‐rich phyllosilicates (Figure 5e,f). Higher‐resolution SEM observations reveal that the black layers of oxide‐dominant *Tubotomaculum* (Figure 4a,b) consist of 2–8 μm spheroids embedded in an irregular matrix (Figure 6a–i), whereas the red layers (Figure 4a) consist of 2–8 μm spheroidal cavities within a similar matrix (Figure 6j). These zones are sharply separated (Figure 6k). The radial dendritic filaments (Figure 4b,d,e) consist of channels tens of microns wide (Figure 6l,m), frequently infilled with phyllosilicates (green arrows in Figure 6l). Phyllosilicate‐filled voids also occur between rice‐shaped grains in the carbonate‐dominant *Tubotomaculum* (green arrow in Figure 6n,o). EDS analyses of both *Tubotomaculum* types reveal high Fe and Mn contents, with minor Ca (Figure S6). Additional elements, such as P, K, Mg, Al, Ba, and Na, are also detected (Figure S6). At the μm scale, both the spheroids and the matrix display a distinctive Mn‐Fe partitioning: spheroids are Mn‐rich, whereas the surrounding matrix is Fe‐rich (Figure 7a–f). This elemental pattern also occurs in the rhombohedral crystals between rice‐shaped grains (MC in Figure 6n,o) of carbonate‐dominant *Tubotomaculum*; these crystals are Fe‐rich but contain Mn‐rich spheroids at their cores (Figure 7g–i,n).

ICP‐MS analysis of rare earth elements *plus* yttrium (REY) shows that oxide‐dominant *Tubotomaculum* have higher ∑REY (up to 134.42 mg/kg) than carbonate‐dominant ones (up to 107.40 mg/kg; Table S1). Both types, however, exhibit positive anomalies for the redox‐sensitive Eu and Ce (Table S1).

### 4.3 Mineral Composition of *Tubotomaculum*

XRPD data collected from the oxide‐rich rims yielded sharp quartz peaks (Tub‐A and Tub‐B; Figure S7) and broad peaks of poor‐ or nanocrystalline Mn‐Fe oxides, allowing identification of goethite [α‐Fe^3+^OOH], birnessite [(Na, Ca, K)(Mn^4+^, Mn^3+^)_2_O_4_·1.5H_2_O], and possibly todorokite and vernadite (Tub‐A in Figure S7). Carbonate‐dominant *Tubotomaculum* consists mainly of siderite [Fe(CO_3_)] and rhodochrosite [Mn(CO_3_)], with minor quartz (Tub‐Ca in Figure S7).

FT‐IR spectra collected from the oxide‐rich rim (Tub‐A and Tub‐B in Figure S8) show bands of goethite at ~894 and ~796 cm^−1^ and quartz at ~1030 cm^−1^. Very broad absorption bands at ~470 and 524 cm^−1^ are assigned to poor crystalline Mn oxides, such as birnessite and todorokite (Potter and Rossman 1979; Bernardini et al. 2019). In agreement with XRPD, the spectrum collected from sample Tub‐Ca shows bands of carbonates (siderite and rhodochrosite) at 727, 867, 1423, and 1808 cm^−1^, and quartz at 1030 cm^−1^. SO_4_^2−^ modes at 619, 1110, and 1193 cm^−1^, combined with Ba‐ and S‐ rich μm‐sized grains in the Fe‐rich matrix (red arrow in Figure S9), indicate trace amounts of barite [BaSO_4_].

**FIGURE 8:**
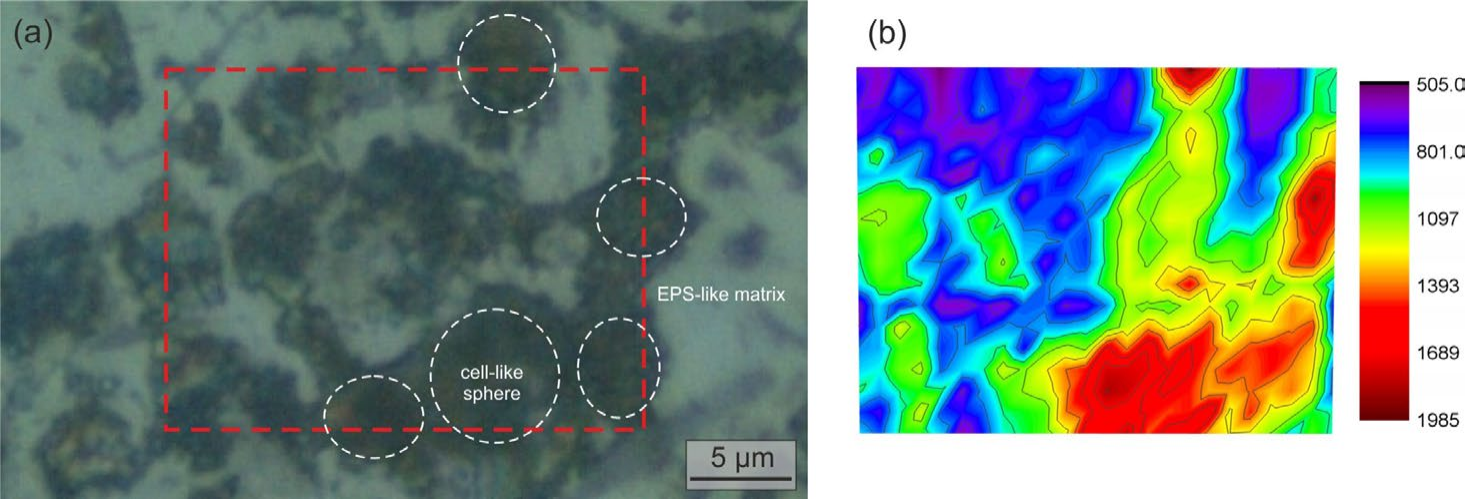
Distribution of carbonaceous remnants at the microbial‐cell scale. Optical image from a black layer (a) and Raman map obtained by integrating the intensity of the G band (~1560 cm^−1^; Figure S11) over the scanned area (dashed white box in a) (b). Red and blue areas: Higher and lower amorphous carbon content, respectively. Laser power 226 μW/μm^2^ mW, exposure time 8 s. White dashed circles: Cell‐like spheres (darker areas) embedded in an EPS‐like matrix (lighter areas).

Raman analyses of oxide‐rich rims (Tub‐A and Tub‐B) yielded two spectra characterized by (1) a strong peak at 645 cm^−1^ and a shoulder at ~573 cm^−1^ (Figure S10a), and (2) peaks at 505, 577, 635, and 735 cm^−1^ (Figure S10b). Integrations of Raman, XRPD, and FT‐IR results allow unambiguous identification of todorokite [(Ca, Na, K)(Mn^4+^, Mn^3+^)_6_O_12_·nH_2_O] and birnessite and/or vernadite (Bernardini et al. 2019). Following Bernardini, Bellatreccia, Della Ventura, and Sodo (2021), Mn occurs both as Mn^4+^ (strong scattering ~640 cm^−1^) and Mn^3+^ (scattering at ~580 cm^−1^; Figure S10). Broad Raman bands at ~1350 and 1560 cm^−1^ (Figure S11) correspond to the D band (disorder in sixfold aromatic rings) and G band (*sp* ^2^ carbon atoms) of amorphous carbon (Ferrari and Robertson 2000). Raman mapping of the G intensity reveals enrichment of amorphous carbon within the Mn‐rich spheroids (red area in Figure 8).

**FIGURE 9:**
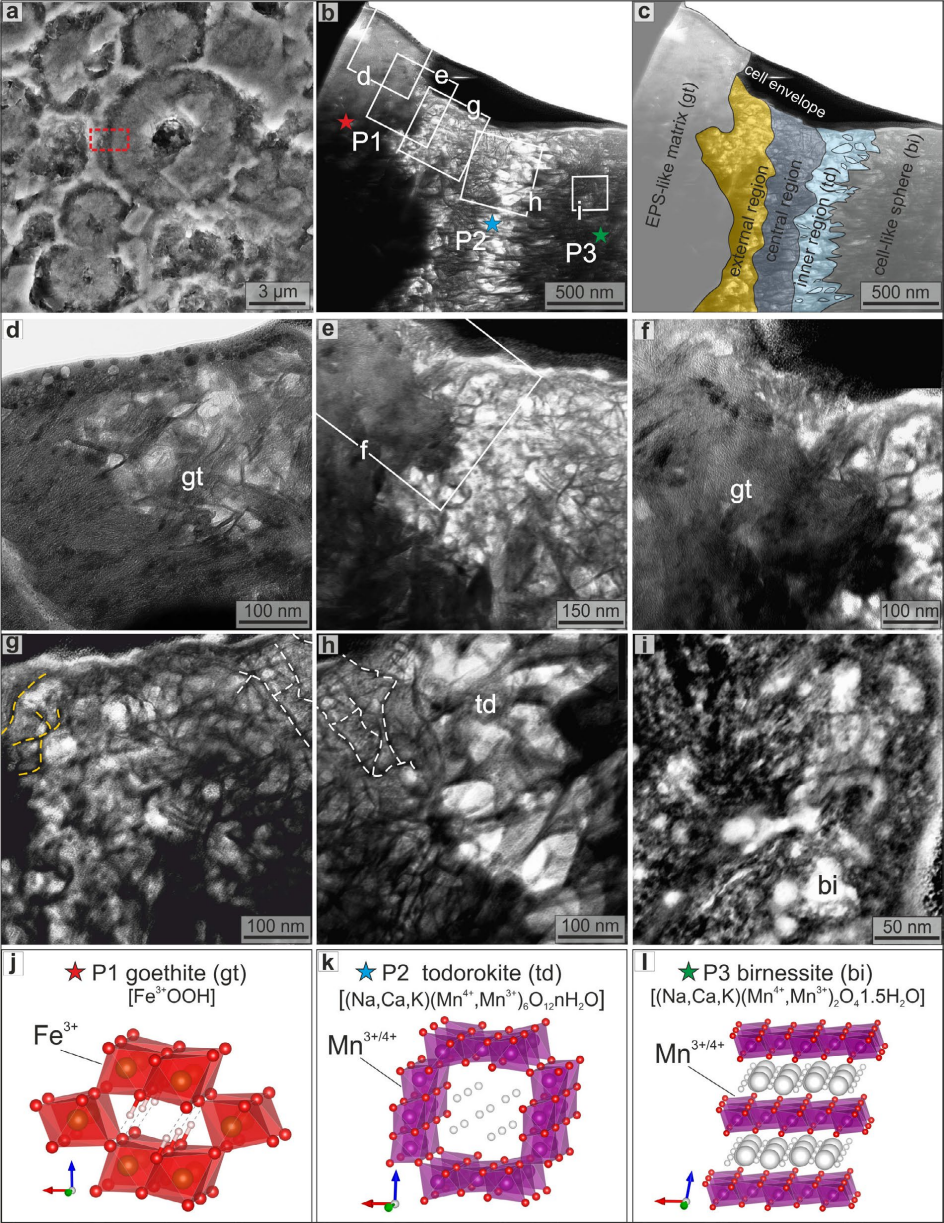
Nanoscale distribution of minerals at the boundary between a cell‐like sphere and the surrounding EPS‐like matrix. SEM image of lamella extraction area (red dashed rectangle) for TEM and ED analyses of an oxide‐dominant *Tubotomaculum* (Tub‐A) (a). TEM image of the boundary between an EPS‐like matrix (Fe‐rich) and a cell‐like sphere (Mn‐rich) (b) and interpretation (c). High‐magnification TEM images of the EPS‐like matrix (goethite) (d–f), the imprint of the cell envelope (external and central regions in c) (g), the todorokite ring (inner region in c) (h), and of the cell‐like sphere (birnessite) (i). Crystal structures of goethite (j), todorokite (k), and birnessite (l). The match between morphologies and distribution of elements and minerals provides evidence that the cell envelope is the redox boundary controlling the separation between Mn and Fe. White and yellow dashed lines: Thread‐like structure and filamentous forms observed in the central region and in the external region, respectively. P1–P3: ED data collection points (Figure S12). gt: Goethite, td: Todorokite, and bi: Birnessite. a, b, and c crystallographic directions are shown as red, green, and blue arrows, respectively. Crystal structure drawing using Vesta (Momma and Izumi 2008).

TEM imaging and polycrystalline electron diffraction (ED) from the sphere‐matrix boundary (Figure 9a) of an oxide‐rich rim (Tub‐A) are given in Figure 9 and Figure S12. Diffraction patterns from the Fe‐rich irregular matrix (P1 in Figure 9b) show rings of nanocrystalline goethite at *d*‐spacing of 5.0, 4.2, 2.7, and 2.4 Å (Figure S12a). In contrast, the Mn‐rich sphere (P3 in Figure 9b) yields birnessite rings at *d*‐spacing of 7.1, 3.6, 2.6, 2.3, 1.7, and 1.4 Å (Figure S12c). Between these zones lies a ~500 nm thick ring (Figure 9a,b) with well‐crystallized tunnel‐structured Mn oxides. ED data from this boundary (P2 in Figure 9b) include a ~10.0 Å spot (Figure S12b) assignable to the strongest reflection of todorokite (Figure 9k), along with two spots at ~3.2 and 1.6 Å compatible with hollandite and/or cryptomelane. At higher magnification, this ring can be further subdivided into three subregions (Figure 9c): (1) a voids‐rich external region with “filamentous forms” of variable thickness (50–400 nm; yellow dashed lines in Figure 9g); (2) a central ~200 nm thick region containing “thread‐like” structure (white dashed lines in Figure 9g,h); and (3) an inner 200–300 nm thick region with todorokite crystals growing radially, lining the cavity (td in Figure 9h).

A complete list of the identified minerals and their spatial distribution is given in Table S2.

### 4.4 Synchrotron‐Radiation μXRF and X‐Ray Absorption Data

**FIGURE 10:**
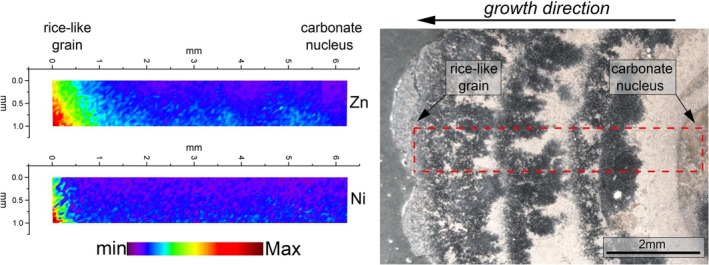
Spatial distribution of Zn and Ni along the growth direction of oxide‐dominant *Tubotomaculum*. SR‐μXRF maps of Zn and Ni from a selected area (red dashed rectangle) of sample Tub‐C. Maps obtained by integrating the intensity of the Kα_1_ of each element over the scanned area. Zn and Ni enrichment during diagenetic precipitation from sediment pore water (Figure S14) mark the transition from hydrogenetic‐to‐diagenetic precipitation during burial beneath the sediment.

The spatial distribution of redox‐sensitive Ni and Zn along the growth direction (from nucleus to surface; black arrow in Figure 10) of an oxide‐dominant *Tubotomaculum* was investigated using SR‐μXRF mapping. The resulting image shows that the black and red layers in the inner portion of the sample are almost devoid of these metals (blue areas in Figure 10), whereas the rice‐like grains at the sample surface are strongly enriched in Ni and Zn (green‐to‐red areas in Figure 10).

The average oxidation state of Mn (AOS) in oxide‐dominant *Tubotomaculum* was determined from SR‐XANES spectra collected from the oxide‐rich rim of sample Tub‐A and from reference‐standard materials (MnO, Mn_2_O_3_, and MnO_2_). The results indicate that Mn occurs predominantly as Mn^4+^ (90%), with a minor Mn^3+^component (10%), yielding an AOS of 3.9 ± 0.15 (Figure S13). The identification of both Mn^4+^ and Mn^3+^ is consistent with Raman spectroscopy results (see Figure S10).

## 5 Discussion

### 5.1 The *Tubotomaculum* Horizon in the Stratigraphic Succession of the Western Mediterranean Basin

Intense back‐arc extension in the Western Mediterranean area during the middle Chattian (~26 Ma) led to the opening of the Algero‐Provençal Basin (Figure 1) (Rosenbaum et al. 2002; Schettino and Turco 2006; Carminati and Doglioni 2012; Savelli 2015; Gómez de la Peña et al. 2021). The seafloor of this nascent ocean was characterized by the deposition of very deep‐water marine sediments (i.e., the Varicolored Clays), accompanied by fluid venting, extreme oxygen depletion, bottom currents of nutrient‐rich waters, and the absence of both infaunal and epifaunal benthic organisms (de Capoa et al. 2007, 2014; Carbone and Grasso 2012; Catalano et al. 2010; Abbassi et al. 2021).

In the Chrabkha section (Tanger, Morocco; Figure 1; Figures S2 and S4), the Varicolored Clays containing the *Tubotomaculum* horizon are ~90 m thick (Abbassi et al. 2021). Given their age interval (~13.4 Ma), they accumulated at a mean sedimentation rate of ~6.7 m/Myr (Figure 2), a value comparable to the ~5.0 m/Myr rate reported by Davies et al. (1977) for upper Oligocene deposits of the Atlantic, Pacific, and Indian oceans.

Previous studies (de Capoa et al. 2007, 2014; Catalano et al. 2010; Carbone and Grasso 2012; Abbassi et al. 2021), as well as our own field observations in the 9th April Dam section and new biostratigraphic analysis, confirm the complete absence of benthic life in the *Tubotomaculum* horizon (Figure 2). Consequently, its age cannot be determined directly. However, the transition from the Varicolored Clays to the overlying Numidian Sandstones is constrained to between 21.0 and 20.5 Ma. This estimate is based on the occurrence of *Sphenolithus delphix* (age range: 23.065–23.328 Ma; Raffi et al. 2006) in a sample collected 14 m below the transition (Abbassi et al. 2021; Figure 2), together with the mean sedimentation rate calculated above.

In the Chrabkha section, as well as in other Tanger‐area localities (e.g., the 9th April Dam section; Figure S4), the *Tubotomaculum* horizon occurs ~30 m below the Varicolored Clays/Numidian Sandstones transition (Figure 2). Applying the sedimentation rate of ~6.7 m/Myr and the constrained age of the transition (21.0–20.5 Ma), we estimate the *Tubotomaculum* horizon to be between 27.0 and 25.0 Ma, thus coeval with the formation of new oceanic crust in both the Ligurian Sea and the Provençal Basin (Figure 1). This correlation suggests that *Tubotomaculum* formed under the environmental conditions that prevailed during the opening of the WMB.

### 5.2 The *Tubotomaculum* Identity: A Long‐Standing Enigma

Over the last 70 years, numerous studies have attempted to clarify the nature of these enigmatic objects. Pautot et al. (1975) demonstrated through XRPD that *Tubotomaculum* consists of rhodochrosite, siderite, and unidentified Mn oxide(s). Based on Mn, Fe, and trace metal (Cu, Ni, and Co) concentrations, they interpreted *Tubotomaculum* as abiotic polymetallic nodules precipitated directly from seawater. This interpretation, however, does not account for their distinctive elongated morphology or their characteristic rice‐like grains (Figure 3).

Earlier, Durand‐Delga (1955) described *Tubotomaculum* from Algeria as pellet‐filled burrows, and this interpretation was later adopted by García‐Ramos (1984) and García‐Ramos et al. (2014) for samples from Alcalá de los Gazules (South Spain). This morphology‐based model was subsequently extended to other Mediterranean occurrences (Riahi et al. 2014; Buatois et al. 2017; Uchman and Wetzel 2017; Menzoul et al. 2022; Naimi and Mahboubi 2025; Spadło et al. 2025). However, several independent lines of evidence challenge this view: (1) the complete absence of benthic life in the *Tubotomaculum*‐bearing horizon (de Capoa et al. 2007, 2014; Catalano et al. 2010; Carbone and Grasso 2012; Abbassi et al. 2021), consistent with an oxygen‐depleted seafloor inhospitable to burrowers; (2) field evidence of reworking at Alcalá de los Gazules (Figure S1), which violates syngenicity and further undermines a trace‐fossil interpretation; and (3) a set of morphological and textural traits absent in true pellet‐filled burrows, including: (i) fusiform, blind‐ended shape (Figure 3); (ii) alternating Fe‐ and Mn‐rich layers surrounding hard nuclei (e.g., bone fragments or silicate/carbonate aggregates; Figure S3c), locally truncated by multiple erosion surfaces leaving relicts of older layers (Figure S3d–f); (iii) microbialite‐like wrinkled laminations (Figure S5); (iv) absence of constructional walls (García‐Ramos et al. 2014); (v) lack of vertically oriented tub‐like structures (García‐Ramos et al. 2014; Spadło et al. 2025; Figure S3); and (vi) anisotropic orientation of rice‐like surface grains (Figure 3).

Spadło et al. (2025) proposed a five‐stage model in which the oxide‐rich layers (red arrows in Figure 4a,b) formed through weathering, either within sediment or after exposure to arid surface conditions. This interpretation, however, fails to account for key internal features, including the systematic occurrence of silicate and bone‐fragment nuclei (Figure 4a,b; Figure S3c), the alternation of fine laminated and massive layers (1 and 2 in Figure S3), and their truncation by a sharp erosion surface (green and red dotted lines in Figure S3d–f). Such sedimentary features, including relict fragments and angular unconformities (Figure S3e,f), are incompatible with inward‐progressing weathering. Instead, they indicate an outward growth process from the nucleus (white arrow in Figure S3), episodically disrupted by erosion, a process closely resembling the formation of marine polymetallic nodules (Hein et al. 2013) and fully consistent with the earlier interpretation of Pautot et al. (1975).

García‐Ramos et al. (2014) alternatively proposed mineralization by secondary Mn‐ and Fe‐rich fluids during early diagenesis. However, this model is contradicted by several independent observations, including the sharp Mn‐Fe separation (Figure 6k), the concentric Fe‐rich (red) and Mn‐rich (black) layers (Figure 5), the systematic presence of hard nuclei (Figure 4; Figure S3c), and the sedimentary features described above (Figure S3d–f). Moreover, field observations show no evidence for secondary Mn‐Fe fluid circulation: no Mn‐Fe‐bearing veins or halos, no geochemical gradients in the surrounding Varicolored Clays (Figures S2 and S3), and no Mn‐Fe veins within the *Tubotomaculum*. Taken together, these inconsistencies render the secondary‐fluid mineralization hypothesis highly unlikely. In addition, if *Tubotomaculum* were pellet‐filled burrows subsequently mineralized, one would expect the presence of Mn minerals typical of diagenetic processes (e.g., 10 Å manganates) and REY patterns or trace‐metal signatures indicative of pore‐water precipitation (Hein et al. 2013; Ortiz Kfouri et al. 2021; Bernardini et al. 2024). However, our results and those of Pautot et al. (1975) indicate a mineralogical and chemical composition characteristic of deposits formed above the sediment–water interface (see below), a setting incompatible with the taphonomic processes expected for burrow fossils (see the schematic model in Figure S14).

Hamoumi (2006) interpreted these structures as corals coated with manganite, hematite, and goethite. However, no coral‐related morphological features were found in our samples (Figures 3 and 4). More broadly, none of the above interpretations adequately explain the formation of Mn oxides under the oxygen‐depleted conditions of the WMB during the middle Chattian.

Given these inconsistencies, a re‐evaluation of *Tubotomaculum* using an integrated stratigraphic, morphological, mineralogical, and geochemical framework is essential to clarify its true nature. Fe‐Mn mineralizations are well known to preserve the biogeochemical conditions existing during growth (Tebo et al. 2004; González et al. 2010, 2012; Hein et al. 2013; Bau et al. 2014; Hein and Koschinsky 2014; Bernardini et al. 2024; Bernardini, Bellatreccia, Columbu, et al. 2021; Ortiz Kfouri et al. 2021; Cornaggia et al. 2020; Basilone et al. 2024), making *Tubotomaculum* a valuable proxy for reconstructing seabed redox chemistry and potential microbiological activity.

Based on stratigraphy, structure, and mineral‐chemical composition, the studied *Tubotomaculum* can be grouped into two types (Table S2): (1) the carbonate‐dominant type, associated with the basal greenish clays, and (2) the oxide‐dominant type, consisting of a hard detrital or carbonate nucleus surrounded by an Fe‐Mn oxide rim, occurring within the reddish clays at the top of the succession (Figure S2). Because greenish and reddish clays typically reflect Fe^2+^‐rich or Fe^3+^‐rich sediments, respectively (Lyle 1983), the upward green‐to‐red color change in the *Tubotomaculum* horizon (Figure S2) marks a Fe^2+^/Fe^3+^ redox boundary. We interpret this shift as the product of changes in bottom‐water ventilation and/or circulation (Jin et al. 2020), which may explain the stratigraphically restricted distribution of two *Tubotomaculum* types.

The concentric Fe‐Mn layers around hard nuclei (red arrows in Figure 4a,b) and the mineral assemblage in oxide‐dominant *Tubotomaculum* (goethite, birnessite/vernadite, and todorokite) are characteristic of deep‐ocean polymetallic nodules (Hein et al. 2013; Hein and Koschinsky 2014). Such nodules typically form by precipitation of Mn‐Fe‐oxides around hard nuclei (rock fragments or biogenic remains, including shark teeth or whale ear bones) on sediment‐covered abyssal plains with low sedimentation rates, at water depths of ~4000–6500 m (Hein et al. 2013; Hein and Koschinsky 2014), conditions that closely match those prevailing in the WMB during the deposition of the *Tubotomaculum*‐bearing Varicolored Clays.

### 5.3 The *Tubotomaculum* Identity: A Mineralogical and Geochemical Perspective

Marine polymetallic nodules are generally classified as hydrogenetic, diagenetic, and hydrothermal (Figure S14), each characterized by distinctive mineral assemblages and trace‐element signatures (Hein et al. 2013; Hein and Koschinsky 2014). Hydrogenetic minerals precipitate directly on the seafloor, incorporating Ce and Co from seawater (Hein and Koschinsky 2014), whereas diagenetic minerals form within soft sediments, taking up Ni and Zn from pore water (Hein and Koschinsky 2014; Ortiz Kfouri et al. 2021; Bernardini et al. 2024). Hydrothermal minerals precipitate near vent sites from mixed seawater–hydrothermal fluids (Hein and Koschinsky 2014).

The rare earth and yttrium (REY) content is a reliable discriminator among nodule types (Bau et al. 2014). Hydrogenetic nodules (cyan line, Figure S15) typically exhibit a positive Ce anomaly and high REY concentrations. Diagenetic nodules (yellow line, Figure S15) show a negative Ce anomaly and intermediate REY contents, while hydrothermal deposits (red line, Figure S15) have negative Ce anomalies, positive Y and Eu anomalies, and generally low REY contents.

Shale‐normalized REY patterns of carbonate‐ and oxide‐dominant *Tubotomaculum* are nearly identical (Figure S15), indicating formation under comparable conditions and suggesting a genetic link. For oxide‐dominant *Tubotomaculum*, both (Ce/Ce*)_SN_ and (Y/Ho)_SN_ values plot in the hydrogenetic field (Figure S16a). Their relatively low REY concentrations compared to typical hydrogenetic nodules (Figure S15) suggest mixing between seawater and hydrothermal fluids, a conclusion further supported by their intermediate position in the (Ce/Ce*)_SN_ vs. Nd diagram (Figure S16b) and by a positive (Eu/Eu*)_SN_ anomaly of up to 1.58 (Table S1).

Carbonate‐dominant *Tubotomaculum* exhibit very strong positive (Ce/Ce*)_SN_ anomalies (up to 1.72; Figure S17; Table S1), consistent with carbonate precipitation under anoxic conditions (Feng et al. 2010). This is in agreement with the greenish color of associated clays (Figure S2) typical of Fe^2+^‐rich anoxic sediments (Lyle 1983). Like the oxide‐dominant type, they also display a strong positive (Eu/Eu*)_SN_ anomaly (up to 1.59; Table S1), indicating precipitation from a mixture of cold seawater and hydrothermal fluids (Bau and Dulski 1996). Additional support for seafloor precipitation comes from ellipsoidal (< 5 μm) barite aggregates (Figure S9), a known product of water‐column precipitation (Paytan et al. 2002).

Our SR‐μXRF maps show surface enrichment in Ni and Zn (Figure 10), consistent with incorporation from pore water during burial, indicating a hydrogenetic‐to‐diagenetic transition (Figure S14) and confirming that *Tubotomaculum* formed above the sediment.

In summary, our dataset demonstrates that *Tubotomaculum* are polymetallic hydrogenetic nodules precipitated from mixed seawater and hydrothermal fluids, remaining exposed on the seafloor until burial. This model is consistent with Pautot et al. (1975) and definitively rules out purely morphological trace‐fossil interpretations (Durand‐Delga 1955; García‐Ramos 1984; García‐Ramos et al. 2014; Riahi et al. 2014; Uchman and Wetzel 2017; Buatois et al. 2017; Menzoul et al. 2022; Naimi and Mahboubi 2025; Spadło et al. 2025). With this framework established, the central challenge is to explain how Mn^2+^ oxidation could occur under the oxygen‐depleted seafloor conditions of the WMB. As discussed below, resolving this paradox offers compelling indications of a microbe‐mediated contribution to the formation of *Tubotomaculum*.

### 5.4 REY Signature of *Tubotomaculum* and Its Implications for Past Microbial Life

Carbonate‐dominant *Tubotomaculum* are significantly enriched in REY (∑REY up to 107.4 mg/kg; Table S1). Such high REY concentrations are commonly observed in microbially mediated marine carbonates (Kamber et al. 2014). In contrast, oxide‐dominant *Tubotomaculum* are relatively depleted in REY (∑REY up to 134.42 mg/kg; Table S1) compared to typical hydrogenetic nodules (Figure S15), consistent with microbially mediated marine polymetallic nodules (González et al. 2010).

Although the formation of deep‐ocean nodules remains debated, several studies suggest that at least some result from biomineralization (Wang et al. 2012; Tully and Heidelberg 2013; Yli‐Hemminki et al. 2014; Blöthe et al. 2015). Such nodules can host different microbial communities that mediate complex redox cycles of metal species (Tully and Heidelberg 2013; Blöthe et al. 2015). Notably, the Mn phases in oxide‐dominant *Tubotomaculum* (birnessite/vernadite and todorokite; Table S2) are well‐known products of microbially mediated Mn oxidation (Tebo et al. 2004). This interpretation is further reinforced by the presence of phosphorus (a key nutrient) in both *Tubotomaculum* typologies (Figure S6) and by RS and SR‐XANES data (Figures S10 and S13), which reveal oxidized Mn species (Mn^3+^ and Mn^4+^) and an AOS of ~3.9 ± 0.15 (Table S2), another hallmark of biomineralization (Tebo et al. 2004).

Microbially mediated Fe^2+^ oxidation typically produces Fe^3+^ colloids that rapidly precipitate as poorly crystalline ferrihydrite (Emerson et al. 2010). In Fe^2+^‐rich systems, ferrihydrite readily transforms into goethite (Yee et al. 2006), consistent with the goethite observed in our samples (Table S2) as the end product of biologically mediated Fe^2+^ oxidation. In carbonate‐dominant *Tubotomaculum*, the presence of siderite and rhodochrosite (Table S2) is also significant, as both minerals are common microbially mediated carbonates in natural marine environments (González et al. 2010, 2012; Naik‐Samant and Furtado 2019; Kamran et al. 2020).

In summary, the mineralogical and geochemical evidence indicates that *Tubotomaculum* is the product of ancient microbial activity.

### 5.5 Morphological and Chemical Imprint of Microbial Populations: From the Cell to the Community Scale

Following this hypothesis, SEM‐EDS and TEM analyses were performed to search for *bona fide* microbial fossils and microbially influenced sedimentary structures. In microbial mats and biofilms, cell envelopes and extracellular polymeric substances (EPS) act as templates and nucleation surfaces, leaving distinctive morphological and chemical biosignatures in the rock record (Cady et al. 2003; Gillen et al. 2023). EDS imaging documents a sharp separation of Mn and Fe (Figures 5 and 7), a redox‐sensitive elemental pattern that coincides with morphological and structural features of matrix‐enclosed microbial populations.

In natural ecosystems, microorganisms form highly organized biofilms. The EPS matrix provides structural integrity, surface adhesion, and key critical functions such as nutrient sorption, electron transfer, and protection (Flemming and Wingender 2010). EPS production is often enhanced in extreme environments, improving microbial survival and metabolic efficiency (Decho and Gutierrez 2017).

Our SEM‐EDS data reveal an irregular Fe‐rich matrix containing cell‐shaped voids that closely resembles fossilized EPS (Figures 6 and 7). In oxide‐dominant *Tubotomaculum*, this matrix, composed of goethite, forms a pervasive network (the red layers in Figure 4). Within the black layers (red arrows in Figure 4a,b), spherical Mn‐infills resembling microbial cells are embedded in the Fe matrix (Figure 7a–c). In carbonate‐dominant *Tubotomaculum*, analogous cell‐shaped rhodochrosite infills occur (Figure 7d–f) in clusters (interpreted as microbial colonies; MC in Figures 4 and 6), separated by rhombohedral siderite (cyan arrows in Figure 4) and phyllosilicates (green arrows in Figure 6n,o). These structures constitute the fundamental building blocks of the mineralization (Figure 3a,c).

TEM and ED data revealed exceptionally well‐preserved ring structures (Figure 9b) between the EPS‐like matrix (goethite, an Fe oxyhydroxide with channel structure; Figure 9j) and the cell‐like spheres. This ring comprises three distinct regions (Figure 9c): (1) a void‐rich external region (50–400 nm thick; yellow dashed lines in Figure 9g); (2) a central region containing filamentous structures (~150 nm thick; white dashed lines in Figure 9g,h); and (3) an inner region of radially oriented todorokite crystals (~250 nm thick; Figure 9h). The microbial cell envelope is composed of a peptidoglycan cell wall (10–100 nm thick), frequently overlain by a crystalline S‐layer (a highly porous protein meshwork 5–70 nm thick) and a capsule (a hydrated matrix composed of polysaccharides or proteins, with a variable thickness of 25–1000 nm) (Silhavy et al. 2010; Pum et al. 2013). These surfaces possess an overall electronegative charge that promotes mineral nucleation (Beveridge 1989; Fortin et al. 1997; Frankel and Bazylinski 2003).

Based on these analogies, we interpret the thick external ring (yellow area in Figure 9c) as the mineralized capsule, the central filamentous region (grey area) as the mineralized S‐layer and cell wall, and the inner todorokite ring (cyan area) as the product of *post‐mortem*, surface‐catalyzed, intracellular oxidation of Mn^2+^. This process would have led to the formation of oriented crystals of a channel‐structured Mn oxide (Figure 9h,k). Subsequent oxidation resulted in birnessite/vernadite nanocrystals, layered Mn oxides (Figure 9l), progressively filling the cell volume either partially or completely (Figure 9i).

These combined morphological, chemical, and mineralogical biosignatures strongly support a microbiological origin for *Tubotomaculum*. When preserved under anoxic conditions, fossil biofilms may retain the carbonaceous remnants of microbial cells and altered biomarker compounds; Fe and Mn minerals, in particular, enhance the long‐term preservation of organic carbon (Estes et al. 2017; Moore et al. 2023). The filamentous structures interpreted as cell‐envelope imprints (Figure 9g,h) could thus retain carbonaceous matter. In fact, RS microanalysis detected amorphous carbon (Figure S11), and RS mapping indicates its concentration within the cell structures (Figure 8), supporting a derivation from degraded cell envelopes. Lipid biomarker analysis (not shown) on a decalcified sample did not yield pristine compounds but showed signatures of secondary inputs.

At the community scale, biofilms consist of clusters of cells separated by a network of anastomosing water micro‐channels and voids that facilitate nutrient delivery and maintain optimal growth conditions (Costerton 1995; Stoodley et al. 2002). During growth, biofilms expand in a fractal fashion into complex branching morphologies, evolving from labyrinthine networks to concentric layers and radial channels, structures that enhance fluid transport and the availability of chemical substrates (Wilking et al. 2013; Zhang et al. 2016; Wang et al. 2022).

Our observations reveal such architectures. In carbonate‐dominant *Tubotomaculum*, elongated microbial‐like clusters (MC in Figures 3, 4, and 6) are separated by a network of open‐water voids (cyan arrows in Figure 4a,c,f–h). The presence of clay minerals (green arrows in Figure 6n,o) and rhombohedral siderite crystals with cell‐shaped rhodochrosite cores (Figure 7g–i) provides evidence of past fluid circulation, which transported clays and altered surface‐exposed mineralized cells.

In contrast, oxide‐dominant *Tubotomaculum* exhibits a more complex rim structure (red arrows in Figure 4a,b), consisting of an inner zone with concentric layers and an outer zone of branching Fe‐rich micro‐channels (Figure 6l,m), represented by red radial filaments (Figure 4d,e; Figure S5). This structural pattern is typical of growing biofilms (Wilking et al. 2013; Zhang et al. 2016; Wang et al. 2022). The detection of calcium within the radial filaments (Figure 5d) and clays infilling the channels and voids (Al‐rich areas in Figure 5e,f; green arrows in Figure 6) provides additional evidence of the past fluid circulation through a living microbial community.

### 5.6 *Tubotomaculum:* Self‐Organized Microbial Populations Shaped by Nutrient‐Rich Bottom Currents

Hydrodynamic stress, nutrient availability, and rheological properties strongly influence the mechanical stability and spatial architecture of biofilms (Stoodley, Doods, et al. 1999; Stoodley, Lewandowski, et al. 1999; Thomen et al. 2017). For example, microbial colonies exposed to turbulent flow tend to elongate downstream, forming migrating ripple‐like structures that promote anisotropic growth (Stoodley, Doods, et al. 1999; Stoodley, Lewandowski, et al. 1999).

Rod‐shaped and elongated microbial colonies are documented from deep‐sea hydrothermal vents (Guezennec et al. 1998). By analogy, we interpret the elongated morphology of *Tubotomaculum* and the anisotropic orientation of the colonies at the biofilm‐seawater interface (MC in Figure 3) as the combined result of branched radial growth and the rheological response of an expanding biofilm to turbulent shear stress generated by nutrient‐rich bottom currents. These currents were likely sustained by localized hydrothermal venting, as supported by our REY data indicating precipitation from mixed seawater‐hydrothermal fluids. This interpretation is reinforced by the isotropic orientation of colonies in the inner portions of the sample and at the biofilm‐sediment interface (Figures 3c,d and 4c), where shear stress was minimal. Comparable genetic processes, linking microbial activity, fluid venting, and bottom‐current influence, have been proposed for the genesis of tube‐like polymetallic and carbonate nodules in the Gulf of Cádiz (González et al. 2010, 2012).

### 5.7 An Integrated Genetic Model for *Tubotomaculum*

Our integrated dataset provides robust evidence that *Tubotomaculum* are mineralized microbial colonies and provide a key to resolving the long‐standing enigma of Mn^2+^ oxidation under the oxygen‐depleted seafloor conditions prevailing during the opening of the WMB.

**FIGURE 11:**
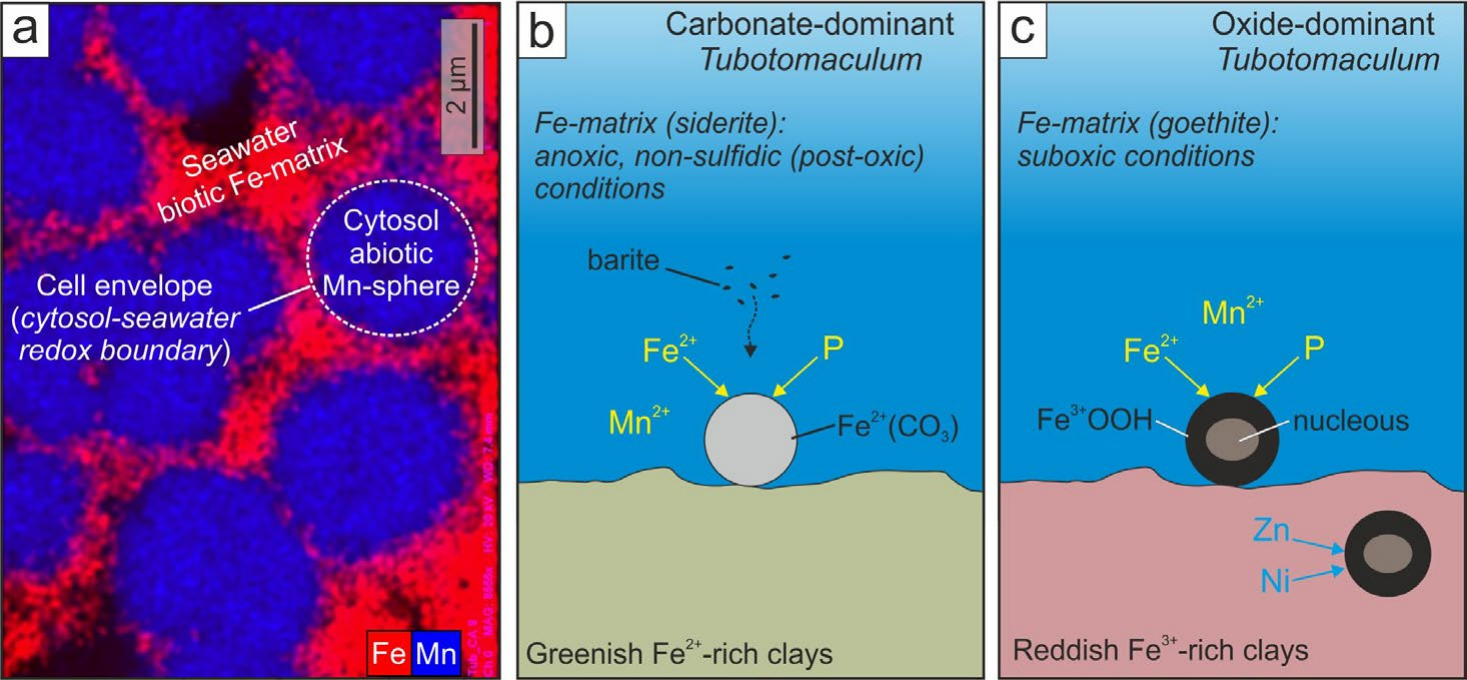
Benthic microbial colonization of the nascent Western Mediterranean Basin. (a) Partitioning of Mn (blue) and Fe (red) between cells and EPS within *Tubotomaculum*, likely driven by the cytosol‐seawater redox contrast. EPS‐mediated Fe minerals (siderite and goethite, red) record ambient seawater conditions during biofilm growth, whereas cytosol‐derived Mn minerals (rhodochrosite and Mn oxides, blue) reflect intracellular conditions. (b) Genetic model for carbonate‐dominant *Tubotomaculum*, formed during deposition of greenish clays under anoxic, non‐sulfidic (post‐oxic) conditions. (c) Genetic model for oxide‐dominant *Tubotomaculum*, formed during deposition of reddish clays under suboxic conditions. Incorporation of marine barite prior to burial, as well as burial‐related enrichment in Zn and Ni from porewaters, is also indicated.

Our model is grounded in four main observations:

1. Mn solubility is significantly higher than that of Fe at pH < 8 (Hem 1972).
2. Fe^2+^ oxidation occurs exclusively outside the cell envelopes (red in Figure 11a).
3. Mn^2+^ oxidation occurs exclusively within the cell envelopes (blue in Figure 11a).
4. Most bacteria and archaea maintain cytoplasmic pH between 7.5 and 7.8, even under extreme environmental conditions (Padan et al. 2005; Slonczewski et al. 2009).

These constraints provide the missing piece of the puzzle: the cell envelope acts as a Mn^2+^/Mn^3+^‐Mn^4+^ redox boundary (white dotted line in Figure 11a), separating an alkaline cytosol (pH ~8), where Mn^2+^ oxidation is favored (blue in Figure 11a), from the surrounding seawater, where ambient redox conditions promote Fe^2+^ oxidation (red in Figure 11a). The cytosol‐seawater contrast explains both the Mn‐Fe partitioning across the cell envelope (Figure 11a) and the occurrence of Mn^2+^ oxidation under extreme oxygen‐depleted seafloor conditions.

At the nanoscale, Fe and Mn minerals at the cell‐EPS boundary (Figure 9) preserve a clear temporal sequence. EPS‐mediated precipitation of Fe‐bearing minerals during active cell metabolism represents a biomineralization phase, with extracellular incorporation of Fe^2+^ into siderite or Fe^3+^ into goethite, forming a protective coating around living cells (red in Figure 11a). The nucleation and radial growth of todorokite crystals with channel structure (Figure 9) at the cell wall in oxide‐dominant *Tubotomaculum* mark the onset of intracellular permineralization, coinciding with microbial death and envelope degradation. Within the alkaline cytosol, permineralization continued with rhodochrosite (Mn^2+^) or mixed‐valence Mn^3+^/Mn^4+^ oxides with a layered structure (birnessite/vernadite; blue in Figure 11a).

Geochemical signatures constrain the seabed environments that hosted these microbial communities: REY contents and associated (Ce/Ce*)_SN_ and (Eu/Eu*)_SN_ anomalies indicate that EPS‐mediated siderite and goethite (red in Figure 11a) formed from mixed seawater–hydrothermal fluids. Siderite forms under anoxic (O_2_ < 10^−6^ mol/L) but non‐sulfidic (H_2_S < 10^−6^ mol/L) conditions, either methanic or post‐oxic (Berner 1981). The δ^13^C values of ~ −10‰ for carbonate‐dominant *Tubotomaculum* (Spadło et al. 2025) match post‐oxic settings (Maynard 1982), where oxygen is consumed by aerobic decay of organic matter but organic flux is too low to sustain high sulfate‐reduction rates (Berner 1981). Because sulfate reduction decreases with decreasing sedimentation rate (Maynard 1982), the limited sulfate reduction recorded in *Tubotomaculum* aligns with the relatively low sedimentation rate of the associated Varicolored Clays (~6.7 m/Myr). Pronounced (Ce/Ce*)_SN_ anomalies (Table S1) further point to carbonate precipitation under anoxia. In this extreme environment, associated with greenish Fe^2+^‐rich clays (Figure S2), Fe^2+^ was incorporated into microbe‐mediated siderite, while Mn^2+^ remained dissolved in the water column (Figure 11b) until cell death, when it was incorporated into rhodochrosite (blue in Figure 11a).

Goethite generally forms under oxic environments (O_2_ ≥ 10^−6^ mol/L; Berner 1981), yet Mn oxides are even more reliable indicators of fully oxic conditions (Froelich et al. 1979). Since Mn^2+^ oxidation in oxide‐dominant *Tubotomaculum* was restricted to the cell interior (blue in Figure 11a), Fe^2+^ oxidation likely occurred under suboxic conditions, consistent with reddish Fe^3+^‐rich sediments (Figure S2; Lyle 1983). In this setting, biofilm growth around hard nuclei (fine‐sediment aggregates or bone fragments on the seabed) produced an EPS‐mediated goethite matrix, while Mn^2+^ persisted in the water column (Figure 11c) until *post‐mortem* incorporation into Mn oxides.

These benthic, self‐organized extremophile microbial communities colonized zones where near‐bottom currents supplied hydrothermal nutrients; the flow likely shaped their fusiform morphology and anisotropic colony orientation at the biofilm‐seawater interface. As the colonies expanded, they progressively accumulated finely laminated to massive Fe‐ and Mn‐rich layers around hard nuclei (oxide‐dominant *Tubotomaculum*). To sustain growth, they developed an internal channel network that enhanced circulation and nutrient transport within the community. In some cases, however, this gradual buildup was interrupted by episodic erosion events, producing sharp breaks in the growth sequence (Figure S3). Phosphorus from continental weathering, recycled at the seafloor during diagenesis (Paytan and McLaughlin 2007), likely sustained the elevated P concentrations in *Tubotomaculum*, supporting biofilm growth at the sediment‐water interface. Marine barite, precipitated within sinking particulate matter (Figure 11b), was incorporated from seawater before burial, while subsequent Zn and Ni uptake from porewaters enriched the rice‐like surface grains (Figure 11c).

In this scenario, microbial colonies in the lowermost greenish clays thrived under post‐oxic conditions, whereas those in the uppermost reddish clays developed under suboxic conditions (Figure 11). The occurrence of distinct *Tubotomaculum*‐types, each associated with a specific stratigraphic position (reddish Fe^3+^‐rich clays vs. greenish Fe^2+^‐rich clays), mineral assemblage (oxides vs. carbonates), and community‐scale architecture (Fe‐Mn layers and radial channels vs. discrete microbial‐like clusters), points to alternating microbial populations that adapted dynamically to shifting environmental conditions in response to redox perturbations in WMB bottom waters.

Exceptional preservation of biosignatures, from cell envelopes to entire biofilm architecture, was likely promoted by: (1) suboxic to post‐oxic bottom waters; (2) Fe^3+^ coating on cell walls (red in Figure 11a), inhibiting organic matter degradation by suppressing autolysis (Ferris et al. 1988); and (3) Ca^2+^‐induced structural biofilm stabilization, enhancing resistance to mechanical dispersion (Nishikawa and Kobayashi 2021).

In summary, our multi‐method study integrates stratigraphic, morphological, mineralogical, and geochemical evidence into a coherent genetic model for *Tubotomaculum*, resolving its long‐standing enigma and revealing new insights into microbial colonization and biosignature preservation under extreme conditions.

## 6 Conclusions and Implications for the Search for Life Beyond Earth

We demonstrate that *Tubotomaculum* are phosphorus‐rich, microbially mediated mineralizations preserving a diverse suite of morphological, chemical, and mineralogical biosignatures characteristic of self‐organized microbial populations. These include: (1) imprints of cell envelopes; (2) cell‐EPS partitioning of biologically relevant redox‐sensitive Mn and Fe; (3) ∑REY values typical of microbial‐mediated marine deposits; (4) oxidized Mn species (Mn^3+^ and Mn^4+^) with AOS of ~3.9 ± 0.15, typical of biomineralization; (5) carbonate and oxide mineral assemblages typical of biomineralization; (6) cluster‐assembled, Mn‐rich microbial cells (colonies) with abundant carbonaceous remnants; (7) an irregular, Fe‐rich, EPS‐like matrix; (8) microbialite‐like and branching structures; (9) a channel network for nutrient transport; and (10) the three‐dimensional biofilm architecture.

These microbial communities thrived under extreme oxygen‐depleted conditions (from post‐oxic to suboxic) that were inhospitable for macrobenthic fauna, particularly burrowers. The stratigraphically restricted distribution of the two *Tubotomaculum* types indicates adaptation to shifting redox regimes. The complete absence of all other forms of benthic life in the *Tubotomaculum* horizon demonstrates that these microbial communities were the earliest colonizers of the inhospitable seafloor of the nascent WMB. As the deep sea represents the largest habitat on Earth, *Tubotomaculum* provides a valuable model for studying past microbial ecology and adaptation to extreme environments.

Beyond their paleoenvironmental significance, our findings show that Fe‐Mn mineralizations have exceptional potential to record biological processes in extreme environments and preserve their signature over geological time. This has profound astrobiological significance: the recent detection of Mn oxides on Mars (Lanza et al. 2014), combined with their strong association with microbial activity on Earth, highlights their value as potential biosignature repositories. For this reason, they represent a high‐priority target for NASA's Mars 2020 and ESA's ExoMars missions. The Raman spectrometer onboard NASA's *Perseverance* rover enables in situ identification of Mn‐Fe mineral phases, Mn oxidation states (Bernardini et al. 2019; Bernardini, Bellatreccia, Della Ventura, and Sodo 2021; Bernardini, Della Ventura, Mihailova, and Sodo 2025; Bernardini, Della Ventura, Sodo, and Mihailova 2025), and potential organic remnants trapped during (bio)mineralization. Such analyses could provide direct evidence of past microbial activity, clarify the biogeochemical evolution of Mars, and guide sample selection for the upcoming NASA/ESA Mars Sample Return mission.

## Conflicts of Interest

The authors declare no conflicts of interest.

## Supporting information


**Data S1:** gbi70031‐sup‐0001‐supinfo.pdf.

**Data S2:** gbi70031‐sup‐0002‐Tables.docx.

## Data Availability

The data that support the findings of this study are available from the corresponding author upon reasonable request.
