# Supplementary material for: The Tubotomaculum Enigma and the Rise of Benthic Life During the Opening of the Western Mediterranean Basin
Source: Geobiology. 2025 Sep 9;23(5):e70031. doi: 10.1111/gbi.70031 (PMC12418153; doi:10.1111/gbi.70031)
Supplement: Supplementary file 1 — Data S1: gbi70031‐sup‐0001‐supinfo.pdf. [file GBI-23-e70031-s002.pdf]

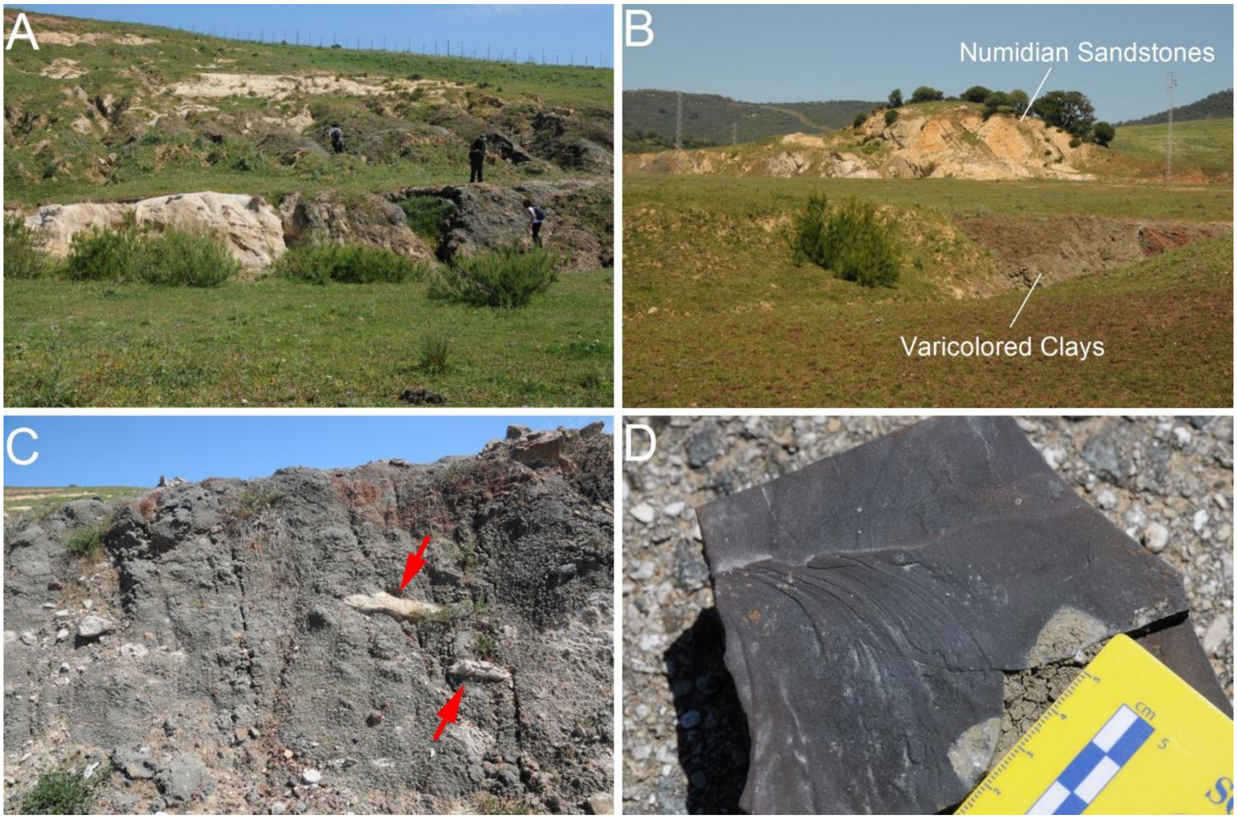

Figure S1. *Tubotomaculum* horizon in South Spain. (A) Panoramic view of the "Arcillas con *Tubotomaculum*" near Alcalá de Los Gazules (see Fig. 1), where blocks of various lithologies and ages are embedded within Varicolored Clays. (B) Block of "Areniscas del Aljibe" (Numidian Sandstones, light tones) embedded within Varicolored Clays; its lowermost part consists of dark clays containing *Tubotomaculum*. (C) White limestone and sandstone blocks (red arrows) wrapped in a varicolored argillaceous matrix hosting *Tubotomaculum*. (D) Allochthonous block of fine-grained sandstone with *Zoophycos* within the "Arcillas con *Tubotomaculum*".

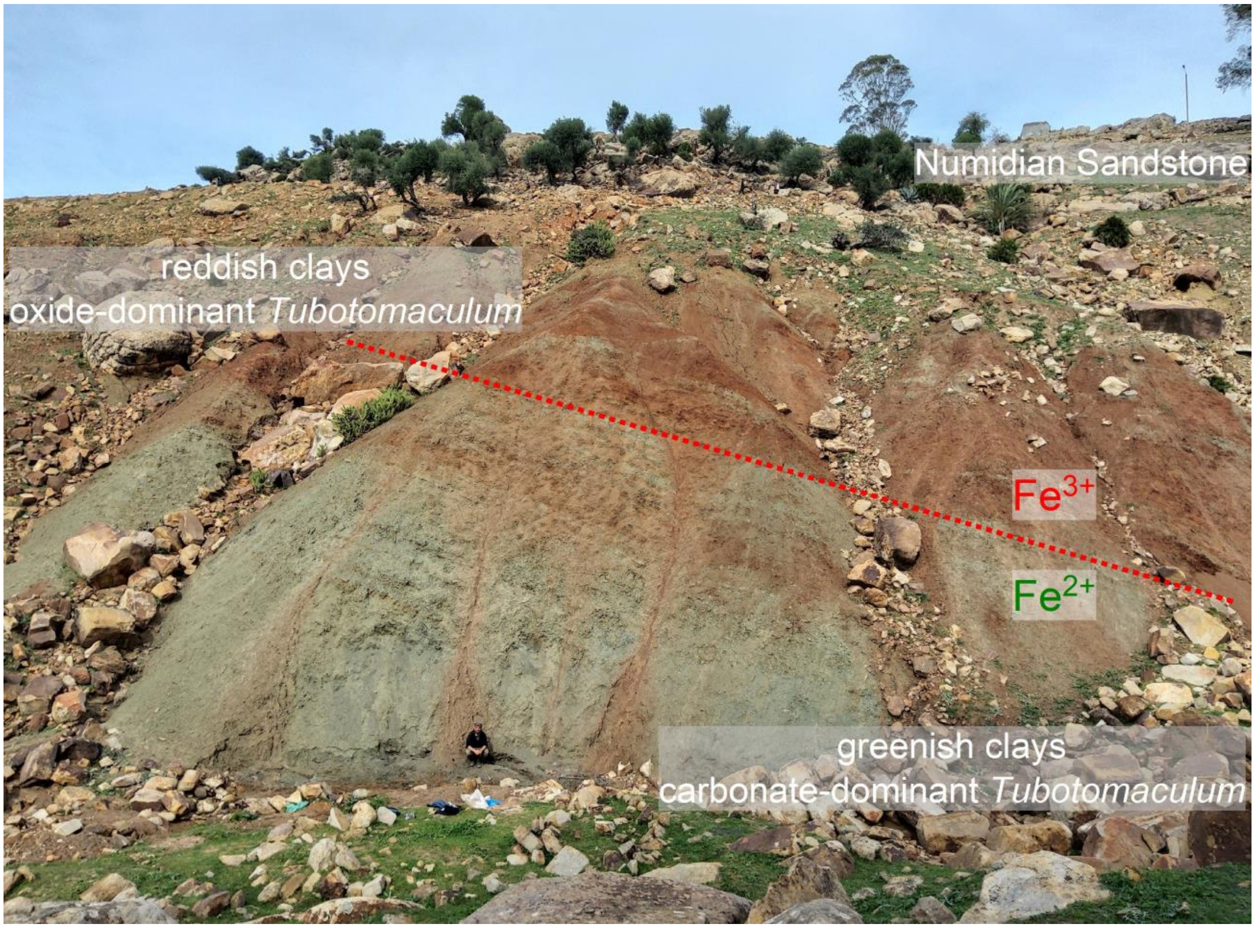

Figure S2. *Tubotomaculum* horizon in Morocco. In the western Rif, the Varicolored Clays form a continuous, undeformed stratigraphic succession without allochthonous blocks. Carbonate-dominant *Tubotomaculum* occur in the lower greenish (Fe^{2+}-rich) clays, whereas oxide dominant samples occur in the upper reddish (Fe^{3+}-rich) clays. The red dashed line marks the Fe^{2+}/Fe^{3+} redox boundary in the *Tubotomaculum* horizon. See Figure S4 for location.

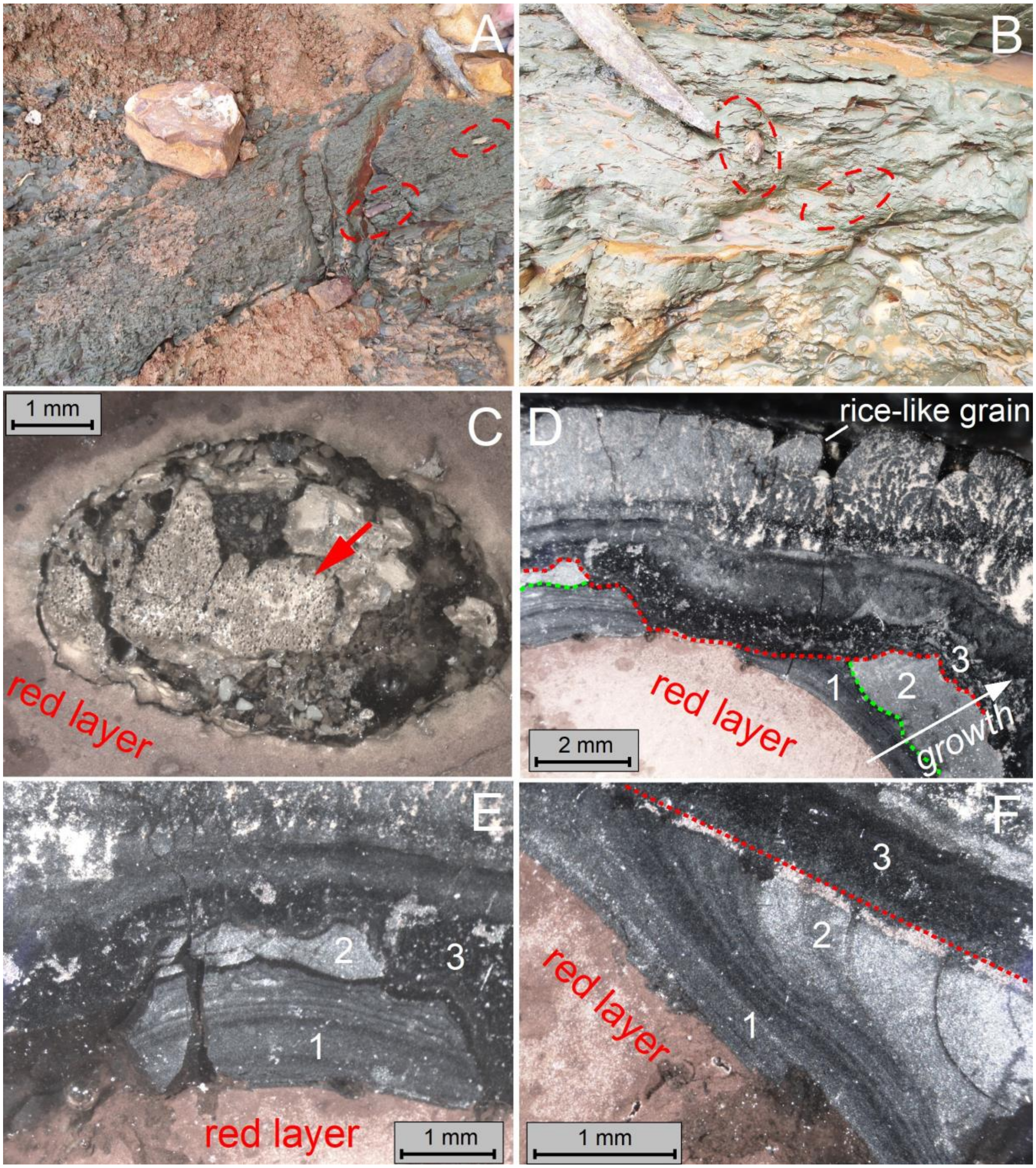

Figure S3. *Tubotomaculum* from the western Rif (Morocco). (A,B) Carbonate-dominant *Tubotomaculum* (red dashed circles) within greenish clays near the Reservoir Lake 9th April (see Figs. S2, S4) as observed in the field. Note the absence of vertical, tube-like bioturbation structures. (C-F) Images of transversal sections collected under the binocular microscope. (C) Nucleus of an oxide-dominant *Tubotomaculum* (Tub-A), consisting of a fragment of a mesh-like type of bone tissue (red arrow) and a fine mixture of silicate minerals. (D) Oxide rim of Tub-A showing multiple erosion surfaces (red and green dotted lines). The first surface (green) separates a fine, laminated blackish layer (1) from a massive grayish layer (2). The second surface (red) truncates both layers as well as the red layer surrounding the nucleus (see panel c). Above this, layer 3 developed, displaying a clear growth sequence from the nucleus outward to the rice-like grains at the sample surface (white arrow). (E) Relict fragments of layers 1 and 2 preserved between the red layer and layer 3. (F) Angular unconformity (red dotted line) where layers 1 (fine laminated) and 2 (massive) are truncated and overlain by layer 3. Together, these internal features, including the presence of hard nuclei and sharp erosion surfaces, stand in strong contrast to a trace-fossil interpretation.

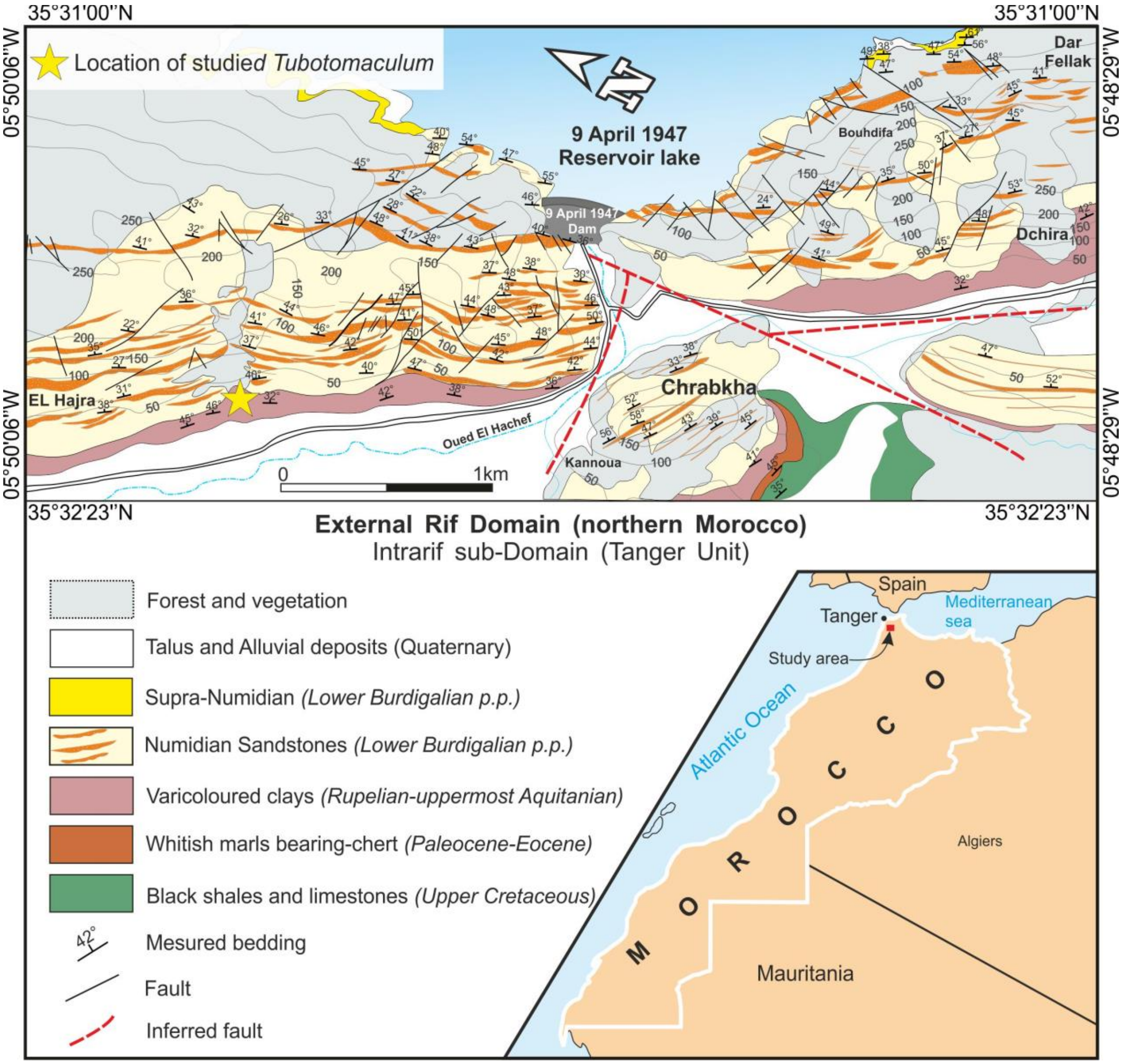

Figure S4. Geological setting and sample location. Detailed geological map of the surrounding area of 9th April 1947 Dam showing the location of oxide-dominant (samples Tab-A, Tub-B, and Tub-C) and carbonate-dominant *Tubotomaculum* (samples Tub-Ca, Tub-Ca1, and Tub-Ca2). The study area is located in the northwestern Rif Chain within the Tanger Unit of the Intrarif sub-domain. In Chrabkha village, a distinctive NE-SW and NW-dipping homocline succession of Tanger Unit croups out. This unit features thin mudstone levels interbedded with Upper Cretaceous black shales. Moving upward, Upper Cretaceous deposits pass to purple marls (Paleocene) and then whitish marls with black chert nodules (Eocene). The whitish marls, perfectly concordant and without any pervasive tectonic deformation, pass up section to reddish and greenish clays (Varicolored Clays). In the upper part, Varicolored Clays contain thin, fine-grained sandstone beds and, about below the top, a *Tubotomaculum*-rich horizon outcrops. Finally, the approximately 1200 m thick Numidian Sandstones overlie the Varicolored Clays, arranged dominantly in wide synclines and anticlines. The lateral continuity of sandstone beds is slightly interrupted by minor strike-slip faults, while major faults displace sectors to the west and east of the dam. After Abbassi et al. (2021).

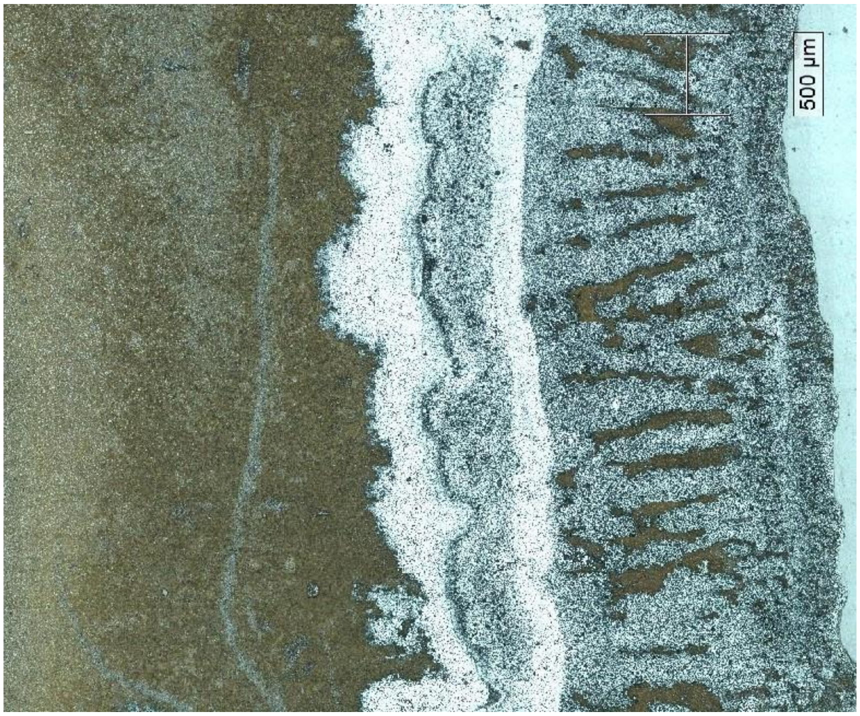

Figure S5. Optical microscopy of microbialite-like structures. OM image of an oxide-dominant *Tubotomaculum* rim showing planar wrinkled laminations (alternating dark/bright laminae in the central area) and radial dendritic filaments (right side area).

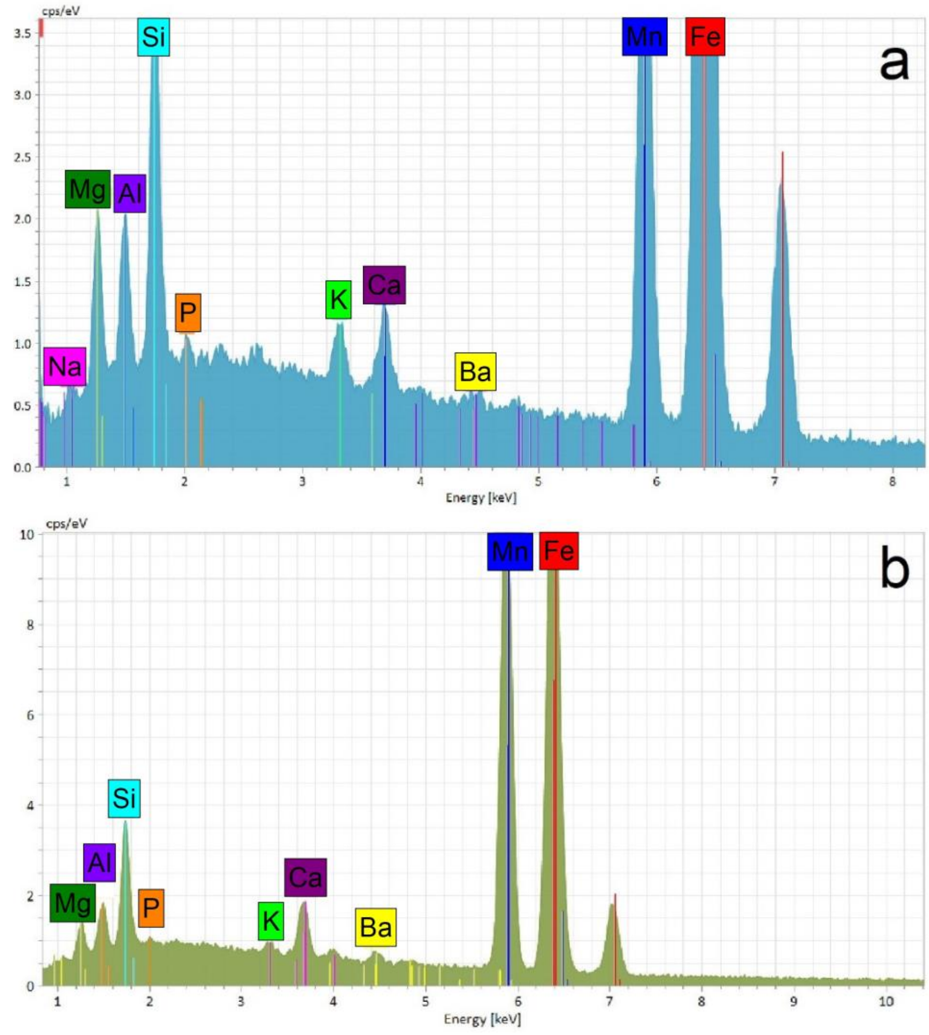

Figure S6. Chemical composition of *Tubotomaculum*. EDS spectra from oxide-dominant rim (Tub-A, Tub-B) (a) and carbonate-dominant *Tubotomaculum* (Tub-Ca) (b).

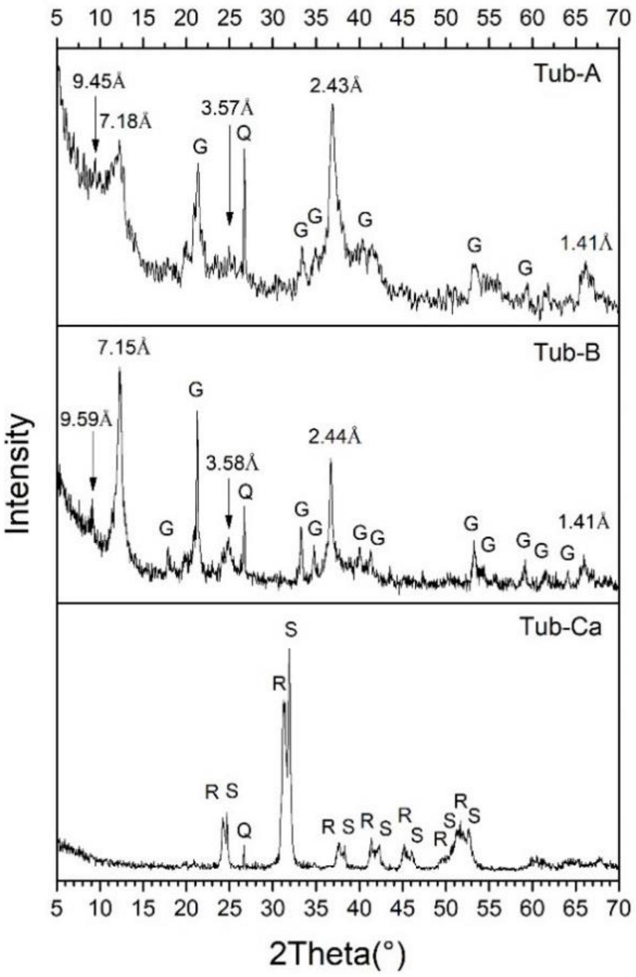

Figure S7. X-ray diffraction analysis of *Tubotomaculum*. XRPD patterns from oxide-dominant rim (Tub-A, Tub-B) and carbonate-dominant *Tubotomaculum* (Tub-Ca). G: goethite, Q: quartz, R: rhodochrosite, S: siderite.

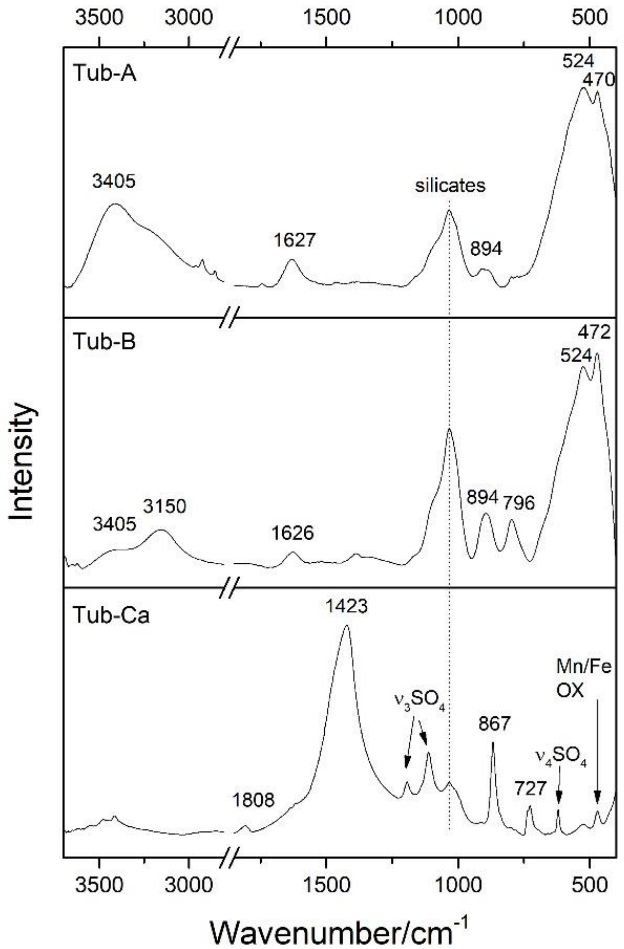

Figure S8. Infrared analysis of *Tubotomaculum*. FT-IR spectra from oxide-dominant rim (Tub-A, Tub-B) and carbonate-dominant *Tubotomaculum* (Tub-Ca).

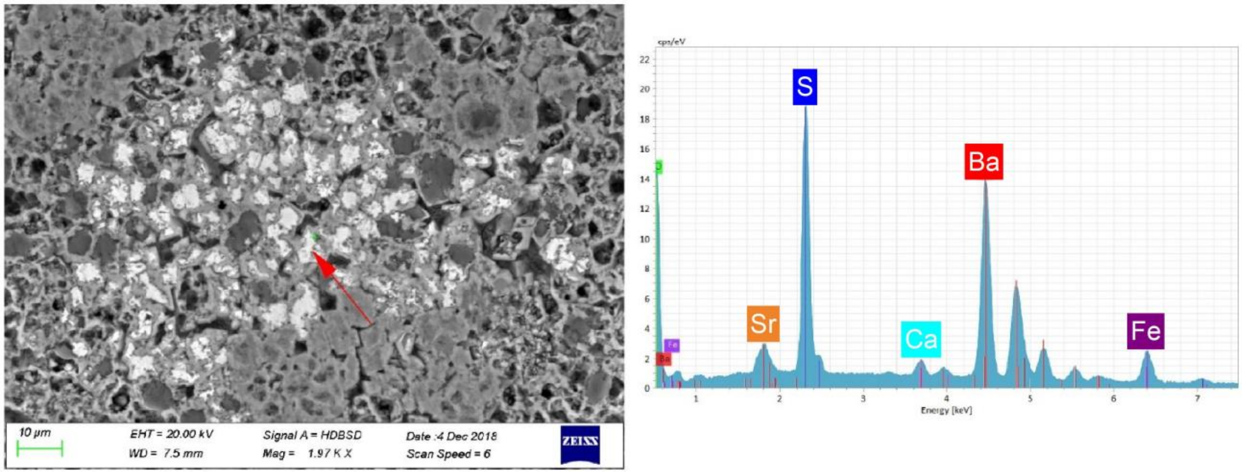

Figure S9. Barite grains in carbonate-dominant *Tubotomaculum*. BSE image and EDS spectrum of barite grains (red arrow) from Tub-Ca.

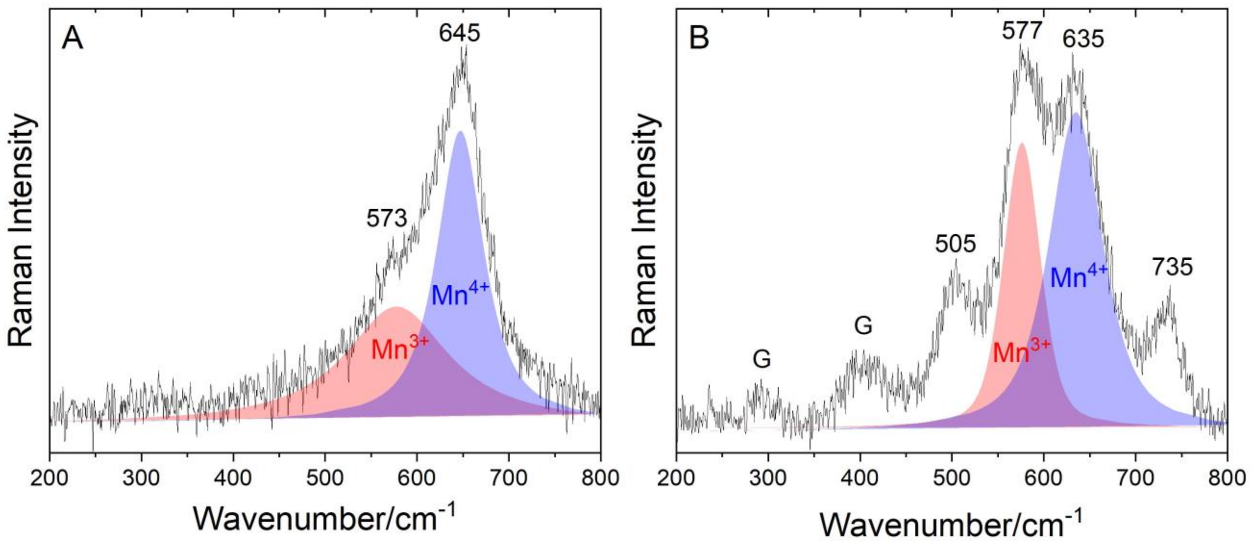

Figure S10. Raman analysis of oxide-dominant *Tubotomaculum*. Raman spectra from oxide-dominant *Tubotomaculum* (Tub-A, Tub-B) showing (A) todorokite and (B) birnessite and/or vernadite. Peaks ~ 575 and 640 cm^{-1} (red and blue) correspond to the ν_1 stretching mode of Mn^{3+}-O and Mn^{4+}-O bonds in Mn-octahedra (Bernardini et al., 2021b). G: goethite.

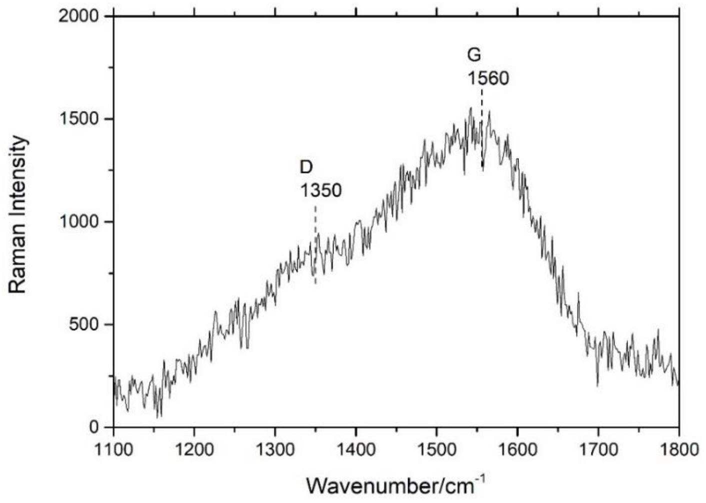

Figure S11. Carbonaceous remnants in *Tubotomaculum*. Raman spectrum from oxide-dominant *Tubotomaculum* (Tub-A) showing the disordered (D) and graphite (G) bands of amorphous carbon.

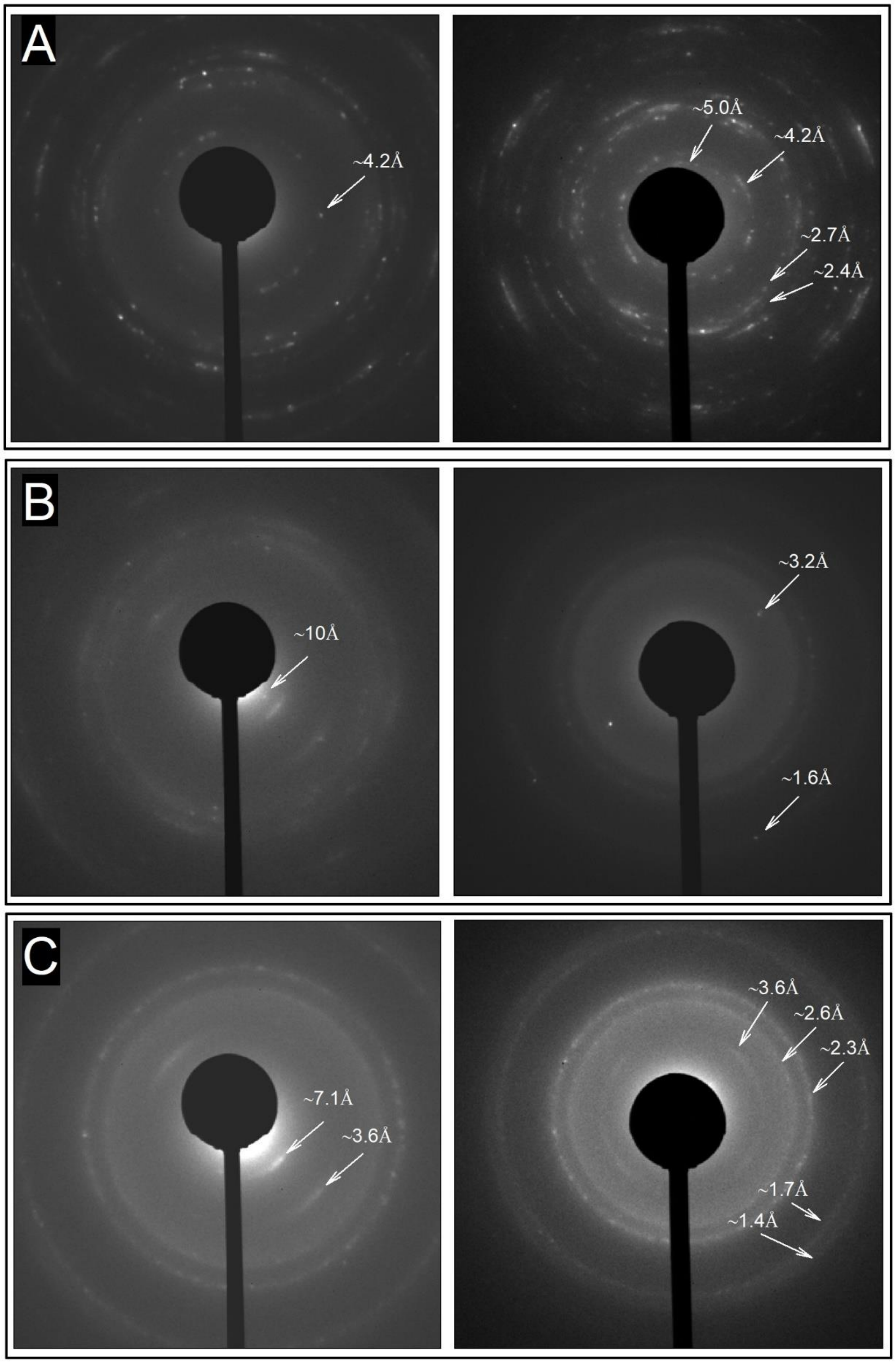

Figure S12. Electron diffraction at the matrix-sphere boundary. ED from oxide-dominant *Tubotomaculum* (Tub-A). (A) Fe-rich matrix (P1 in Fig. 9B); (B) sphere-matrix boundary (P2 in Fig. 9B); (C) Mn-rich sphere (P3 in Fig. 9B).

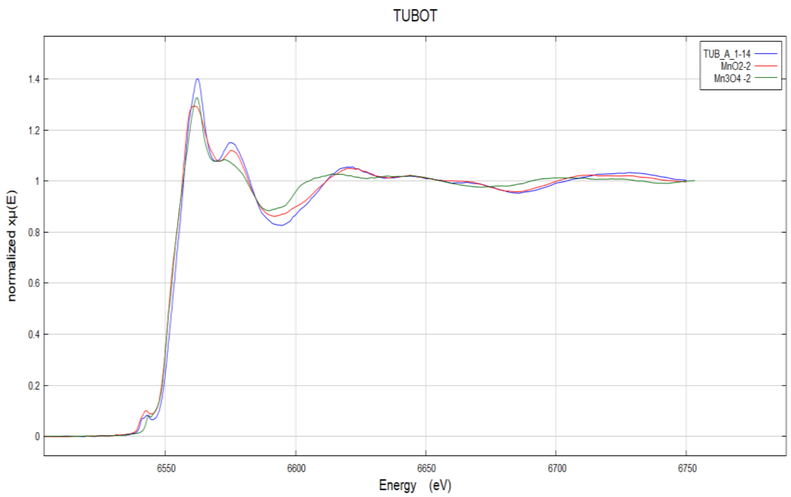

Figure S13. Mn oxidation state from SR-XANES. Spectra from an oxide-dominant rim (Tub-A, blue line) compared with MnO_2 and Mn_3O_4 standards.

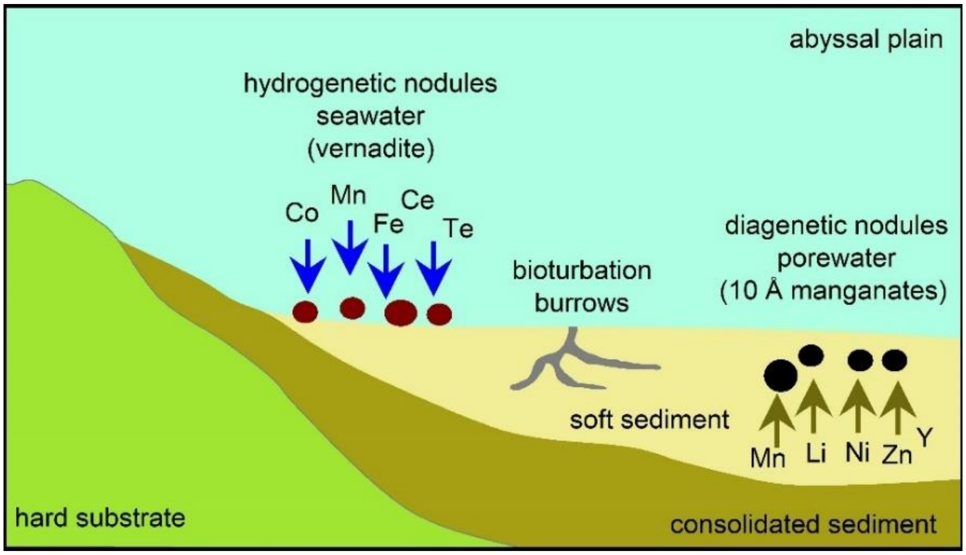

Figure S14. Diagram of formation of deep-ocean polymetallic nodules. Schematic environments of formation of hydrogenetic (vernadite) and diagenetic (10 Å manganates: lithiophorite, asbolane, buserite, todorokite) nodules by precipitation from seawater or sediment porewater, respectively. Note that the mineralization of pellet-filled bioturbation burrows should produce minerals (10 Å manganates) and chemical signatures (REY patterns and trace metals content) typical of diagenetic growth from porewater (Ortiz Kfouri et al., 2021). The elements typically enriched in each growth mode are indicated. Blue and brown arrows indicate accretion from seawater and from porewater, respectively. Modified from Hein et al. (2013).

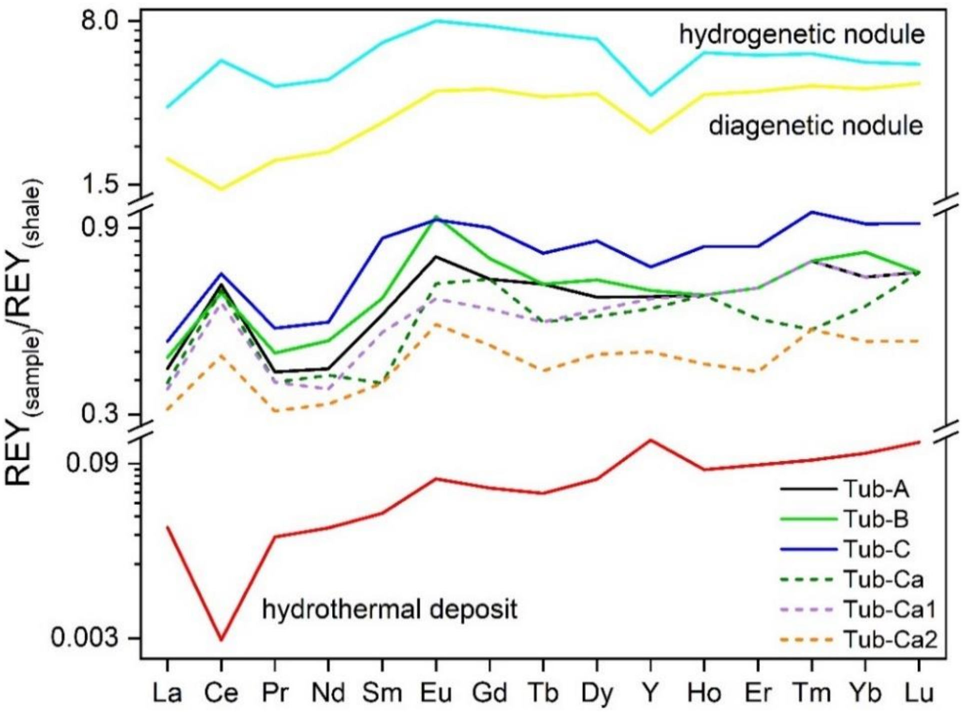

Figure S15. ICP-MS analysis of *Tubotomaculum*. Shale-normalized REY patterns of oxide-dominant (Tub-A, Tub-B and Tub-C) and carbonate-dominant (Tub-Ca, Tub-Ca1 and Tub-Ca2) *Tubotomaculum* (see Table S1). The data for marine hydrothermal deposits (red line), diagenetic nodules (yellow line), and hydrogenetic nodules (cyan line) from Bau et al. (2014). The shale is the Post-Archean Australian Shale (PAAS) from McLennan (1989). The partitioning of REY during the precipitation of Mn-Fe mineralizations is accompanied by strong fractionation between solid and solution and by distinctive fractionation within the REY group (Bau et al., 2014).

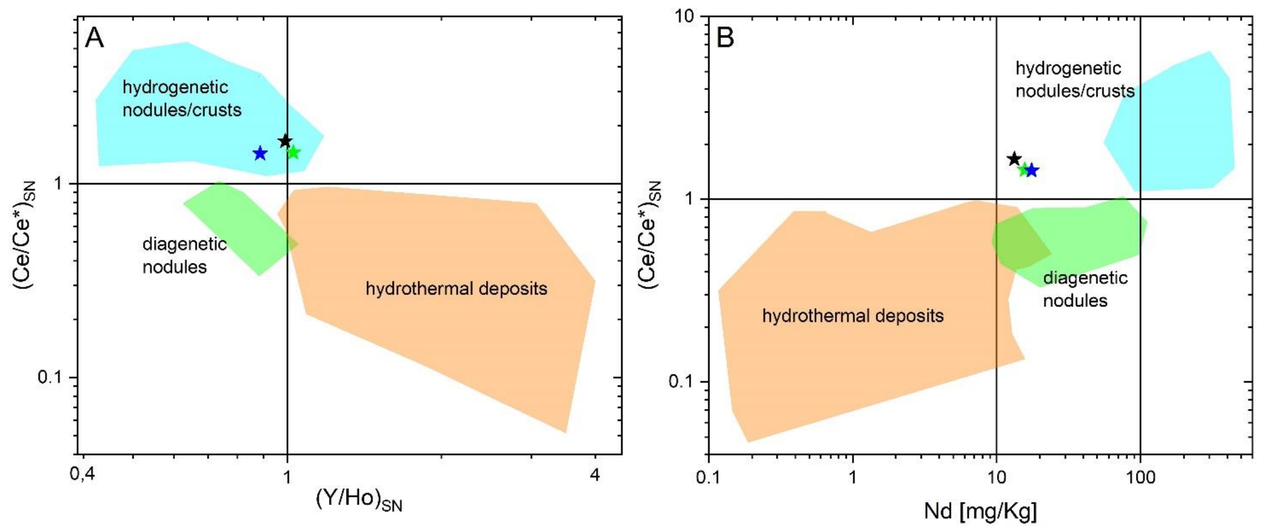

Figure S16. Discrimination diagrams for marine MnFe deposits. (A) $(Ce/Ce^*)_{SN}$ *versus* Y_{SN}/Ho_{SN} and (B) of $(Ce/Ce^*)_{SN}$ *versus* Nd concentration for the oxide-dominant *Tubotomaculum*; after Bau et al. (2014). $(Ce/Ce^*)_{SN} = Ce_{SN}/(0.5La_{SN}+0.5Pr_{SN})$. SN: shale normalized; the shale is the Post-Archean Australian Shale (PAAS) from McLennan (1989). Tub-A: black star, Tub-B: green star, Tub-C: blue star.

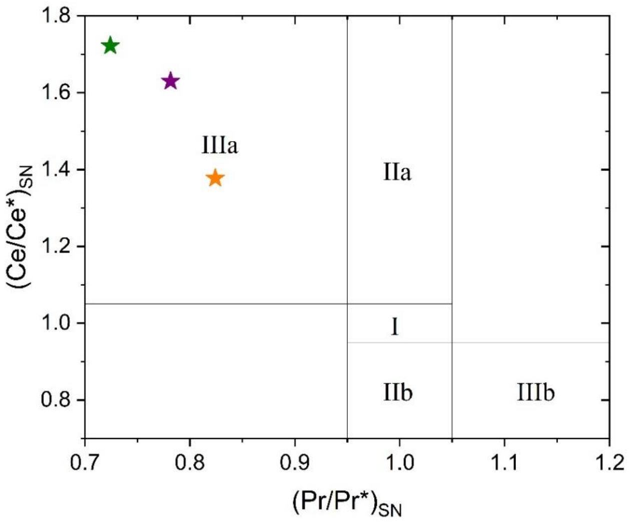

Figure S17. Cerium anomalies in carbonate-dominant *Tubotomaculum*. Plot of $(Ce/Ce^*)_{SN}$ *versus* $(Pr/Pr^*)_{SN}$ for carbonate-dominant *Tubotomaculum*; after Bau and Dulski (1996). Field I: no anomaly; Field IIa: positive La anomaly, no Ce anomaly; Field IIb: negative La anomaly, no Ce anomaly; Field IIIa: real positive Ce anomaly; Field IIIb: real negative Ce anomaly. Note that carbonate-dominant *Tubotomaculum* show real positive Ce anomalies (field IIIa) suggesting precipitation under anoxic conditions. $(Pr/Pr^*)_{SN} = Pr_{SN}/(0.5Ce_{SN} + 0.5Nd_{SN})$ and $(Ce/Ce^*)_{SN} = Ce_{SN}/(0.5La_{SN}+0.5Pr_{SN})$. SN: shale normalized; the shale is the Post-Archean Australian Shale (PAAS) from McLennan (1989). Tub-Ca: green star, Tub-Ca1: purple star, Tub-Ca2: orange star.

References:

Abbassi, A., Cipollari, P., Zaghloul, M.N., Cosentino D., 2021. The Numidian Sandstones in northern Morocco: Evidence for early Burdigalian autochthonous deposition on top of the Tanger Unit. Mar. Pet. Geol. 131, 105149. https://doi.org/10.1016/j.marpetgeo.2021.105149

Bau, M., Dulski, P., 1996. Distribution of yttrium and rare-earth elements in the Penge and Kuruman iron-formations, Transvaal Supergroup, South Africa. Precambrian Res. 79, 37–55.

Bau, M., Schmidt, K., Koschinsky, A., 2014. Discriminating between different genetic types of marine ferro-manganese crusts and nodules based on rare earth elements and yttrium. Chem. Geol. 381, 1–9.

Bernardini, S., Bellatreccia, F., Della Ventura, G., Sodo, A., 2021b. A reliable method for determining the oxidation state of manganese at the microscale in Mn oxides via Raman spectroscopy. Geostand. Geoanal. Res. 45, 223–244.

Hein, J.R., Mizell, K., Koschinsky, A., Conrad, T.A., 2013. Deep-ocean mineral deposits as a source of critical metals for high- and green-technology applications: comparison with land-based resources. Ore Geol. Rev. 51, 1–14.

McLennan, S.M., 1989. Rare earth elements in sedimentary rocks; influence of provenance and sedimentary processes. Rev. Mineral. Geochem. 21, 169–200.

Ortiz Kfouri, L., Millo, C., Estela de Lima, A., Silveira, C.S., Sant'Anna, L.G., Marino, E., González, F.J., Sayeg, I.J., Hein, J.R., Jovane, L., Bernardini, S., Lusty, P.A.J., Murton, B.J., 2021. Growth of ferromanganese crusts on bioturbated soft substrate, Tropic Seamount, northeast Atlantic ocean. Deep Sea Res., Part I 175, 103586. https://doi.org/10.1016/j.dsr.2021.103586
