# Supplementary material for: The Tubotomaculum Enigma and the Rise of Benthic Life During the Opening of the Western Mediterranean Basin
Source: Geobiology. 2025 Sep 9;23(5):e70031. doi: 10.1111/gbi.70031 (PMC12418153; doi:10.1111/gbi.70031)
Supplement: Supplementary file 2 — Data S2: gbi70031‐sup‐0002‐Tables.docx. [file GBI-23-e70031-s001.docx]

**Table S1. REY composition of *Tubotomaculum*.** (Eu/Eu*)_SN_ is calculates as Eu_SN_/[(Tb_SN_×Sm_SN_^2^)^1/3^] while (Ce/Ce*)_SN_ as Ce_SN_/(0.5La_SN_+0.5Pr_SN_). SN: shale normalized; the shale is the Post-Archean Australian Shale (PAAS) from McLennan (1989).

|  | Oxide-dominant *Tubotomaculum* | | | Carbonate-dominant *Tubotomaculum* | | |
| --- | --- | --- | --- | --- | --- | --- |
|  | Tub-A | Tub-B | Tub-C | Tub-Ca | Tub-Ca1 | Tub-Ca2 |
|  | (mg/Kg) | | | | | |
| Y | 16.2 | 16.8 | 19.3 | 15.1 | 16.0 | 11.7 |
| La | 15.0 | 16.0 | 17.6 | 13.8 | 13.3 | 11.8 |
| Ce | 51.3 | 48.9 | 54.7 | 49.6 | 46.1 | 33.7 |
| Pr | 3.4 | 3.8 | 4.4 | 3.2 | 3.2 | 2.7 |
| Nd | 13.3 | 15.7 | 17.5 | 12.8 | 11.8 | 10.8 |
| Sm | 3.0 | 3.3 | 4.7 | 2.0 | 2.7 | 2.0 |
| Eu | 0.82 | 1.04 | 1.02 | 0.7 | 0.64 | 0.55 |
| Gd | 3.1 | 3.5 | 4.2 | 3.1 | 2.6 | 2.1 |
| Tb | 0.5 | 0.5 | 0.6 | 0.4 | 0.4 | 0.3 |
| Dy | 2.8 | 3.1 | 3.9 | 2.5 | 2.6 | 2.0 |
| Ho | 0.6 | 0.6 | 0.8 | 0.6 | 0.6 | 0.4 |
| Er | 1.8 | 1.8 | 2.3 | 1.5 | 1.8 | 1.1 |
| Tm | 0.3 | 0.3 | 0.4 | 0.2 | 0.3 | 0.2 |
| Yb | 1.9 | 2.2 | 2.6 | 1.6 | 1.9 | 1.3 |
| Lu | 0.3 | 0.3 | 0.4 | 0.3 | 0.3 | 0.2 |
| ∑REY | 114.32 | 117.84 | 134.42 | 107.4 | 104.24 | 80.85 |
| (Eu/Eu*)_SN_ | 1.32 | 1.58 | 1.15 | 1.59 | 1.19 | 1.38 |
| (Ce/Ce*)_SN_ | 1.66 | 1.45 | 1.43 | 1.72 | 1.63 | 1.38 |

**Table S2. Summary of the identified minerals and their spatial distribution.** Mineral phases in the different Tubotomaculum-type mineralizations, their distribution between the irregular Fe-rich matrix (EPS-like) and the embedded Mn-rich (cell-like) spheres, and dominant Mn species. All data derived from the integrated results of OM, SEM-EDS, XRPD, ED, SR-XANES, FT-IR, and RS analyses

| Sample | Type | Description | Major  minerals | Minor minerals | Irregular  Fe-matrix | Embedded Mn-spheres | Dominant  Mn species |
| --- | --- | --- | --- | --- | --- | --- | --- |
| Tub-A  Tub-B | oxide | detrital* or carbonate nucleus | siderite, rhodochrosite | quartz, barite† | siderite | rhodochrosite  (2-4 µm size) | Mn^2+^ |
|  |  | oxide external rim | goethite, birnessite / vernadite, quartz | todorokite | goethite | birnessite / vernadite, todorokite  (2-8 µm size) | Mn^3+,4+^ |
| Tub-Ca | carbonate | carbonate | siderite, rhodochrosite | quartz, barite† | siderite | rhodochrosite  (2-4 µm size) | Mn^2+^ |

* Detrital nuclei consist of mixtures of phyllosilicate minerals, quartz, and/or bone fragments (Figure S3c). Note that marine polymetallic nodules form by precipitation of Mn-Fe compounds about hard nuclei. The latter are thus crucial to the formation of nodules. † Traces detected only by FT-IR.
